# Spatial Indicators to Monitor Land Consumption for local Governance in Southern Germany

**DOI:** 10.1007/s00267-021-01460-3

**Published:** 2021-03-22

**Authors:** Markus A. Meyer, Isabella Lehmann, Otmar Seibert, Andrea Früh-Müller

**Affiliations:** 1 Research Group on Agricultural and Regional Development, Reitbahn 3, D-91746 Weidenbach, Germany; 2 ITC, University of Twente, Hengelosestraat 99, NL-7514 AE Enschede, Germany

**Keywords:** Land consumption, Land take, Land management, Indicators, Spatial planning, Governance

## Abstract

Land consumption for settlement and infrastructure development has been extensively discussed and analyzed in the last two decades. In Germany, existing governance at the state level seems to hardly foster effective land management at the municipal level to achieve overarching goals at the level of the European Union such as “no net land take”. Germany aims to limit land consumption to less than 30 ha per day by 2030. This goal is hardly translated to the municipal level where actual land-use decisions are taken due to the municipal planning sovereignty. In order to address these deficiencies, this study characterizes land consumption in the Nuremberg Metropolitan Region with self-organizing maps and identifies major factors explaining cluster differences using boosted regression trees. We identified four major clusters: booming, prosperous, moderate, and transition regions. Generally, beneficial demographics (population growth and lower old-age dependency ratio) and financial power of municipalities come at the expense of considerable settlement and traffic infrastructure development (i.e., increased land consumption), creating the impression of a rather unregulated market despite the existing planning framework in Germany. Based on these clusters, we developed an indicator set through a participatory process to improve land-use planning following three dimensions: efficient land use, preservation of cultural landscapes and its services, and fostering the regional added value of agricultural products beyond the current local political focus. Future research should assess whether municipalities with better information will reduce land consumption due to increased awareness.

## Introduction

Land consumption is a major object of study in spatial planning, land science, and related disciplines such as economics (Kment 2018; Nuissl and Schroeter-Schlaack 2009). Land consumption is defined as the conversion of natural or agricultural land to built-up land and its patterns have been extensively described and quantified (e.g., Salvati et al. 2018). The additionally used term “land take” spatially aligns with the term land consumption in spatial planning (Marquard et al. 2020) and is the scope of this study. Internationally, land consumption as defined by UN Habitat (2020) also includes agricultural or forest land use (Marquard et al. 2020).

At the international level, the political relevance of land consumption is reflected in, for instance, the Sustainable Development Goal (SDG) 11 (“Make cities and human settlements inclusive, safe, resilient and sustainable”), which considers the indicator of sustainable land consumption relative to population growth (target 11.3.1) (UN 2015). Notwithstanding, an international study with a European focus classifies land take and land consumption as underrated problems (Marquard et al. 2020).

National targets in Germany aim at reducing land consumption from 56 ha per day in 2018 to 30 ha per day in 2020 (Umweltbundesamt 2020). This target has not been achieved and is now under revision and reconsideration for 2030, in line with the aforementioned SDG (UN 2015). Derived targets for the German federal state of Bavaria demand for a decline from 10 ha (2018) (StMUV 2020) to 5 ha per day in 2030 (StMWi 2020). These land consumption targets at the national or federal level hardly translate into locally targeted impacts due to their nonbinding character and the planning sovereignty of German municipalities. Therefore, a reduction in land consumption is only achievable through the self-commitment of local authorities. Even if single municipalities make efforts to reduce their land consumption, other municipalities might try to gain by attracting land users from land-saving municipalities. In this way, efficiency gains in land consumption in booming regions are counteracted by inefficiencies in other regions, most likely due to lower land prices in regions with low-density urban sprawl (Henger and Bizer 2010; Mascarenhas et al. 2019). This leakage in land consumption provides a difficult governance setting if land consumption for settlement and traffic infrastructure is to be reduced. For the governance of land consumption, an effective and accepted approach in practice is therefore needed.

As a considerable part of municipalities’ financial resources originates from commercial and property tax, the financial strength and fiscal stability of German municipalities depend on residential and commercial area development as shown for Bavaria by Langer and Korzhenevych (2018) (see also Henger and Bizer 2010). To address externalities of land consumption, several ideas for regulation, market interventions, and informational instruments have been proposed. For each of these policy instruments, governance deficits exist (see Henger and Bizer (2010) for detailed explanations). With respect to regulation, the German federal building code relates to the constitution and secures municipalities’ autonomy with respect to (urban) land-use planning. This political setting defines the opportunities for legally binding restrictions to land consumption for municipalities as the responsible bodies for land-use planning. Other authors study and propose market interventions (e.g., Juerges et al. 2018) such as tradable permits for land consumption as a way to internalize societal costs (Henger and Bizer 2010). This has been tested for numerous municipalities in Germany, revealing a considerable decline of land consumption outside the existing settlement areas (Meub et al. 2017). Similarly, Nuissl and Schroeter-Schlaack (2009) propose a Pigouvian tax to internalize the external costs of land consumption, but this has not been empirically tested so far (Juerges et al. 2018) and can be argued as being insufficiently spatially targeted to internalize negative local externalities. Nevertheless, the implementation of both market interventions – tradeable permits or a Pigouvian tax – is unlikely given the missing political agreement across German federal states. Therefore, the third option, informational instruments might be a feasible one with impact in the short or medium term.

To learn from the past and to improve future land-use governance, decision-makers need access to relevant information on the impacts of planning at the municipal level (e.g., Colantoni et al. 2016). A regional monitoring system could enable stakeholders, planners, and politicians to assess the current state of land consumption and its impacts in individual municipalities, compare municipalities with each other, and provide information for inter-municipal benchmarking. Ideally, this could initiate positive competition by revealing municipalities with beneficial and less beneficial land-use planning. Currently, most municipalities are in a prisoner dilemma, which forces them to offer land to private and commercial builders (Henger and Bizer 2010) because if they do not, neighboring municipalities would offer land for development. A regional monitoring system could support inclusive landscape governance by revealing the impacts of land consumption and thus enabling a stakeholder dialog based on quantitative information on the current planning practice (see Bürgi et al. 2017 for a framework on the integrated landscape approach).

To understand and govern land consumption through a monitoring system, land consumption should be seen within the larger context of landscape change and in relation to other land-use/land-cover change pathways. Hence, major interactions with other land-use/land-cover types need to be considered. Existing studies have analyzed landscape-change patterns and drivers across Europe (Kuemmerle et al. 2016; Plieninger et al. 2016). Typically, studies on land consumption examine patterns, relevant drivers, and impacts (e.g., Nuissl et al. 2009 and Salvati et al. 2018). Patterns of land consumption and their drivers have been studied in multiple contexts with different foci (see Behnisch et al. (2018) for an overview for Germany). For example, Kretschmer et al. (2015) discuss the relevance of socio-demographic, economic, and political drivers for land consumption. In addition, several studies quantify the impact of these factors on land-use/land-cover change or on land consumption (Kroll and Haase 2010; Meyer and Früh-Müller 2020). With respect to land consumption patterns, a study in Germany was able to confirm the expected positive relationship between surface or soil sealing^1^ and settlement density with linear regression techniques (Behnisch et al. 2016). However, the study was hardly able to confirm the hypothesized relationship between surface sealing and land consumption patterns (e.g., living space per capita, accessibility of commercial facilities), landscape aesthetics, or topographic characteristics. Hence, further analysis at the sub-national level is needed given the considerable regional heterogeneity in economic development. For example, Germany’s reunification backlog demand for settlement and infrastructure development in eastern Germany, as well as differences in spatial planning (e.g., due to the federal structure) or biophysical conditions require regional studies, as partly revealed in the spatial clustering of error terms of the chosen regression techniques (eastern vs. western, northern vs. southern Germany) (Behnisch et al. 2016). Therefore, a study like this one at the subnational level may provide more reliable insights into the drivers and impacts of land consumption. However, the usually chosen regression techniques hardly consider non-linear relationships and are less optimized for considering cumulative effects and interactions between variables. Following Levers et al. (2018b), this study, therefore, uses boosted regression trees to assess the impact of drivers on land consumption to overcome some of the shortcomings of the study by Behnisch et al. (2016).

Hersperger et al. (2017) propose an indicator set to assess the impact of spatial planning on biodiversity, natural resources, recreation, and landscape aesthetics, as well as economic competitiveness. Although the chosen indicator set considers major environmental impacts, it disregards other relevant categories such as rural development and narrows economic competitiveness to agriculture, forestry, and tourism. It is, therefore, necessary to extend the proposed indicator set to include other relevant impacts of land consumption. Moreover, the analysis of spatial planning and governance (e.g., agricultural and forest policy) in Hersperger et al. (2017) strongly deviates from EU policies such as the Common Agricultural Policy (European Commission 2021). In this context, agricultural land is especially important as it is mainly consumed for settlement and infrastructure development (Azadi et al. 2011). Subsidies (e.g., direct payments) strongly affect the economic viability of farming and land prices, but do not seem to halt agricultural land-use change in Germany (Destatis 2019; Meyer and Früh-Müller 2020).

Against this background, this study aims at (i) characterizing landscape change with an emphasis on land consumption through major clusters with self-organizing maps (Skupin and Agarwal 2008), (ii) identifying major factors that explain cluster differences using boosted regression trees, and (iii) developing indicators to improve the governance of land consumption at municipal level considering results from (i) and (ii). In the next section, we first introduce the specific context of the Nuremberg Metropolitan Region as well as the methods applied in this paper. Nest, we examine the results of the cluster analysis and identify suitable indicators. Finally, we discuss our findings and their implications for regional governance and present the main conclusions.

## Methods

### Study Area

The Nuremberg Metropolitan Region is located in the northeast of the federal state of Bavaria in southern Germany, covers an area of approximately 21,800 km², and has about 3.5 million inhabitants (EMN 2018). European Metropolitan Regions are a spatial category and regional planning concept. There are 11 European Metropolitan Regions and each European Metropolitan Region is a coalition of cities and their surrounding areas. Of the eleven European Metropolitan Regions, the study area is the fourth biggest and has the lowest population density (~163 inhabitants per square kilometer). With around 12%, the share of land used for settlement and transport infrastructure is comparatively low, while the share of afforested land is the highest of all European Metropolitan Regions (Destatis 2020).

### Land-Use/Land-Cover Change Clustering

For the analysis of land-use/land-cover change, we used the major land-cover categories and land-use information for built-up land following the structure of the German official real estate cadaster information system and the Bavarian Office of Statistics (Table 1). We identified patterns of comparable land-use/land-cover change with the variables in Table 1 for the Nuremberg Metropolitan Region using self-organizing maps with *R* (R Development Core Team 2018) and the package *kohonen* (Wehrens and Buydens 2007). Self-organizing maps have been used to identify land-use/land-cover change patterns as they combine the capabilities of principal component analyses and K-means clustering (i.e., reduced dimensionality combined with clustering) (Dittrich et al. 2017). We standardized these land-use/land-cover change variables using z-score normalization (zero mean and unit variance) to remove differences in units. We followed Levers et al. (2018a) and Dittrich et al. (2017) and tested hexagonal output planes from (2 × 2 to 5 × 6) for 4–30 clusters. We selected the final cluster based on the Davies–Bouldin index, which aims at the optimal ratio of intra- and intercluster variability, and based on the sum of the distance of the grid cells to the codebook vectors (Davies and Bouldin 1979; Dittrich et al. 2017). We ran the iterative self-organizing maps algorithm 400 times to obtain stable clustering patterns.

**Table 1:** Variables to quantify land-use/land-cover change patterns for the Nuremberg Metropolitan Region (2008–2018)

Variables	Code	Resolution/scale	References
Land-use/land-cover composition [% municipality]		municipality	BayLfStat 2020f, 2020g, 2020h
*Settlement- and transportation*	SAT		
*Agricultural land*	AG		
*Forest*	FOR		
*Living area*	LIV		
*Commercial area*	COM		
Living space [m²]	LIVS	municipality	BayLfStat 2020i
Residential buildings [*n*]	LIVB	municipality	BayLfStat 2020i

We selected boosted regression trees to determine the major differences between the four major land-use/land-cover change clusters in the Nuremberg Metropolitan Region. We preferred boosted regression trees over traditional regression approaches as they are not bound to the distribution of both dependent and independent variables (Breiman 2001; Elith et al. 2008). The tool *gbm.step* from the package *dismo* (Hijmans et al. 2017) in R Development Core Team (2018) was used to distinguish the clusters. Following Levers et al. (2014) and Meyer and Früh-Müller (2020), we conducted a sensitivity analysis for the combination of learning rates (0.00025–0.01) and tree complexities (1–9) with the tenfold cross-validated correlation coefficient as a quality criterion. Column and row averages were calculated to select the highest combination for further analysis (see Tables S2–S7 in the supplementary material). The model was split into two equal subsamples of training and test data. We calculated the relative contribution of each explanatory variable and used partial dependency plots to interpret the impact of individual independent variables on land-use/land-cover change patterns or cluster separation. For visualization, we smoothed the partial dependency plots and only selected variables above the expected explanatory contribution (Müller et al. 2013), i.e., 100 divided by 39 variables or 2.56 %. Similarly, we visualized the three major interactions between explanatory variables in the supporting information (Figs. S12–S15). The variables to distinguish land-use/land-cover change clusters in Table 2 were selected from recent studies exploring land-use/land-cover change in Central Europe (Behnisch et al. 2016; Hersperger et al. 2017; Kienast et al. 2015; Meinel et al. 2019; Meyer and Früh-Müller 2020).

**Table 2:** Explanatory variables to explain differences of land-use/land-cover change clusters

Variables	Code	Resolution/ scale	References
*Demographic*			
Population development (2008–2018) [%]	POP_08_18	Municipality	BayLfStat 2020b
Population development (2018–2028) [%]	POP_18_28	Municipality	BayLfStat 2020b, 2020c
Old-age dependency ratio (2008–2018,2018) [%]	AQ_08_18, AQ_18	Municipality	BayLfStat 2020a
Old-age dependency ratio (2018–2028,2028) [%]	AQ_18_28, AQ_28	Municipality	BayLfStat 2020a, 2020d
Population density (2018,2028) [*n* ha^−1^]	POP_DENS_18, POP_DENS_28	Municipality	BayLfStat 2020b, 2020g
*Infrastructural*			
High-speed internet (16 Mbit s^−1^) share (2017) [%]	HS_INTERNET_17_P_CAP	Municipality	BBSR 2018
Public transport departures per inhabitant (2018) [%]	PUB_TRANS_18_P_CAP	Municipality	BayLfStat 2020b; BBSR 2018
Supermarkets per inhabitant (2017) [%]	SUP_MARKT_17_P_CAP	Municipality	BayLfStat 2020b; BBSR 2018
Pharmacies per inhabitant (2017) [%]	PHARM_17_P_CAP	Municipality	BayLfStat 2020b; BBSR 2018
Higher education institutions per inhabitant (2017) [*n* n^−1^]	HIGH_EDU_17_P_CAP	Municipality	BayLfStat 2020b; BBSR 2018
Universities per inhabitant (2017) [*n* n^−1^]	UNIVERSITIES_17_P_CAP	Municipality	BayLfStat 2020b; BBSR 2018
*Economic*			
Employees at the place-of-residence (2008–2018) [%]	SO_SEC_LIV_08_18	Municipality	BayLfStat 2020l
Employees at the place-of-work (2008–2018) [%]	SO_SEC_WOR_08_18	Municipality	BayLfStat 2020l
Employees at the place-of-residence (2018) per inhabitant [*n* n^−1^]	SO_SEC_LIV_P_CAP_18	Municipality	BayLfStat 2020b, 2020l
Employees at the place-of-work (2018) per inhabitant [*n* n^−1^]	SO_SEC_WOR_P_CAP_18	Municipality	BayLfStat 2020b, 2020l
Commuter balance (2008–2018) [%]	COMMUTE_BAL_08_18	Municipality	BayLfStat 2020m
Commuter balance per inhabitant (2018) [*n* n^−1^]	COMMUTE_BAL_P_CAP_18	Municipality	BayLfStat 2020b, 2020m
Property Tax A (2008–2018) [%]	PROP_TAX_A_08_18	Municipality	BayLfStat 2020j
Property Tax A per inhabitant (2018) [€ n^−1^]	PROP_TAX_A_P_CAP_18	Municipality	BayLfStat 2020b, 2020j
Property Tax B (2008–2018) [%]	PROP_TAX_B_08_18	Municipality	BayLfStat 2020j
Property Tax B per inhabitant (2018) [€ n^−1^]	PROP_TAX_B_P_CAP_18	Municipality	BayLfStat 2020b, 2020j
Gross commercial tax (2008–2018) [%]	COM_TAX_GROSS_08_18	Municipality	BayLfStat 2020j
Gross commercial tax per inhabitant (2018) [€ n^−1^]	COM_TAX_GROSS_P_CAP_18	Municipality	BayLfStat 2020b, 2020j
Net commercial tax (2008–2018) [%]	COM_TAX_NET_08_18	Municipality	BayLfStat 2020j, 2020k
Net commercial tax per inhabitant (2018) [€ *n*^*−*1^]	COM_TAX_NET_P_CAP_18	Municipality	BayLfStat 2020b, 2020j, 2020k
Financial power (2008–2018) [%]	FIN_POW_08_18	Municipality	BayLfStat 2020e
Financial power per inhabitant (2018) [€ n^−1^]	FIN_POW_P_CAP_18	Municipality	BayLfStat 2020b, 2020e
Tax power (2008–2018) [%]	TAX_POW_08_18	Municipality	BayLfStat 2020n
Tax power per inhabitant (2018) [€ n^−1^]	TAX_POW_P_CAP_18	Municipality	BayLfStat 2020b, 2020n
*Biophysical*			
DEM [m]		50 m	Bayerische Vermessungsverwaltung 2019
Aspect [°]	ASPECT		
Curvature [score]	CURVATURE		
Elevation [m]	ELEVATION		
Roughness	ROUGHNESS		
Slope [%]	SLOPE		
Soil Quality Rating	SQR	250 m	ZALF 2013
Drought index (2004–2009) [mm °C^−1^]	DROUGHT	1 km	DWD Climate Data Center 2018

### Identifying Indicators to Monitor Land Consumption

Based on extensive literature analysis of existing monitoring schemes (see supplementary material, Table S1), data analysis, and expert workshops, a comprehensive set of indicators for a land-use monitoring system was formulated and subsequently condensed. Based on desired objectives of political and development goals, we identified qualitative and quantitative reference values respectively. The set consists of (a) core indicators that can be derived from routinely gathered data sources (e.g., land-use/land-cover or census data), and (b) advanced indicators that are assessed either using spatial models or are derived from surveys at the municipal level.

## Results

### Clusters of Land-Use/Land-Cover Change

**Fig. 1:**
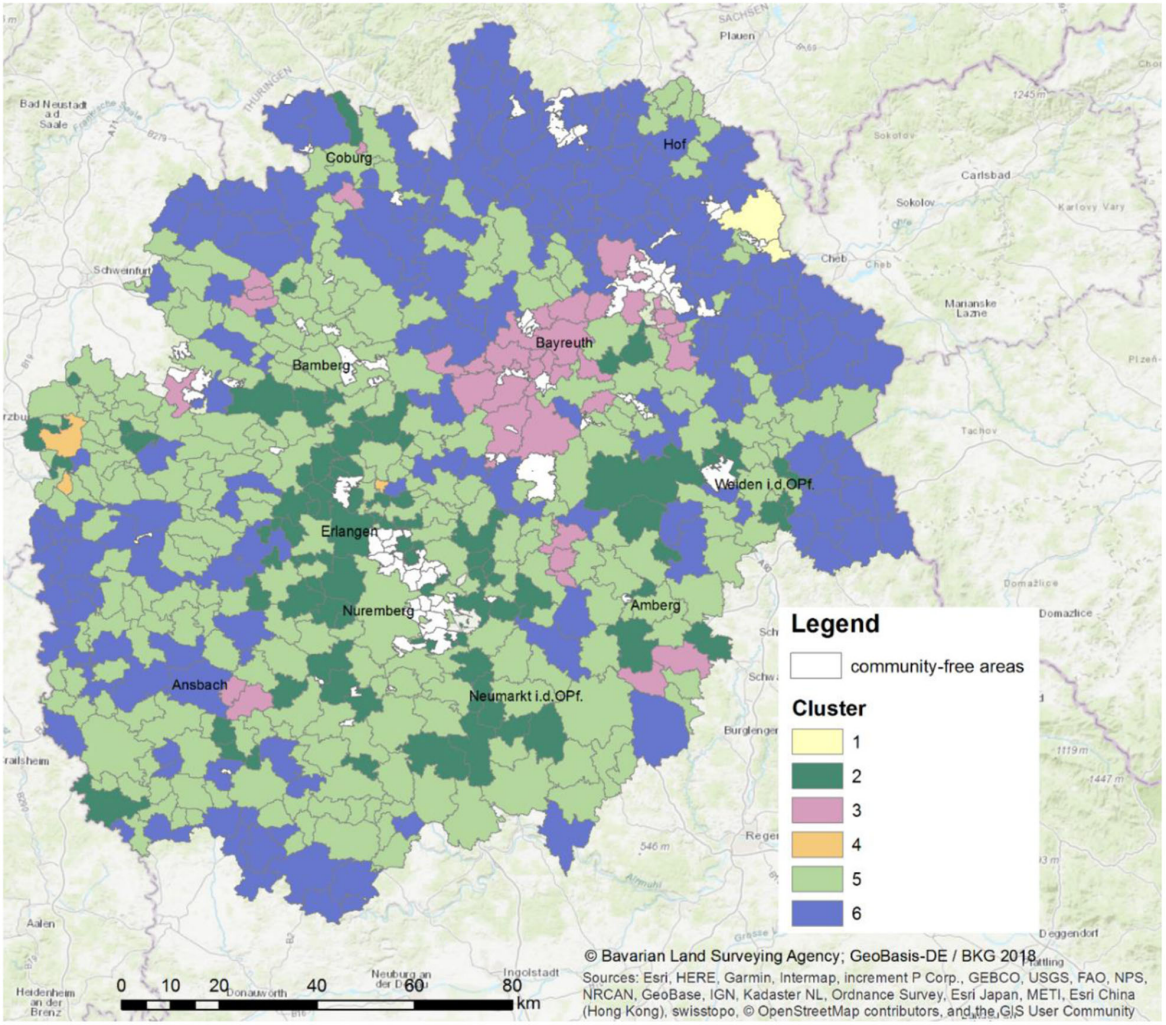
Major land-use/land-cover change clusters of municipalities in the Nuremberg Metropolitan Region (2008–2018) encompassing Cluster 2 “Booming regions”, Cluster 3 “Transition regions”, Cluster 5 “Prosperous regions”, and Cluster 6 “Moderate regions”. Community-free areas are not governed by local municipalities, but administered by a higher authority (e.g., the military or the state forest)

Using the Davies–Bouldin index for an optimal ratio of intra- and intercluster variability (see Fig. S1 in the supplementary material), we identified six clusters of different land-use/land-cover change patterns in the Nuremberg Metropolitan Region for the period 2008 until 2018. Clusters 1 and 4, both consisting of a few municipalities, were outliers. Cluster 1 has an above-average increase in forest area and a considerable decline in agricultural land and settlement area. This outlying municipality was recently joined with a previous forested non-communal area. Cluster 4 is an outlier due to a very strong increase in the commercial area mostly due to new renewable energy developments such as solar power panels. Four major clusters with respect to land-use/land-cover change patterns have been identified: booming, transition, prosperous and moderate regions (Table 3). We named the clusters according to the underlying socio-demographic and economic factors driving land-use/land-cover change. Booming regions were characterized by the strongest increase in residential area development and were mostly located in rural areas along with urban development hotspots in the urban triangle Nuremberg-Fuerth-Erlangen toward Bamberg in the north and between Bayreuth, Weiden, and Amberg in the east of the study area (Fig. 1). Transition regions were characterized by considerably higher settlement area development, comparable residential area development, and a considerably lower increase in residential buildings and buildings area space compared with booming and prosperous regions. Agricultural land decreased most by about 7% and forest increased most by about 3% compared to 2008. Commercial areas slightly declined. Hotspots of this cluster were in the southwest (Franconian Switzerland) and northeast of Bayreuth (Fichtel Mountains), which are most likely structurally weaker and agriculturally less beneficial than other regions in the study area (Fig. 1). Prosperous regions reflected the dominant cluster of the study region with a considerable increase in living space and residential buildings and a slight increase (0.1%) in commercial areas. The major difference to booming regions is the considerably lower increase in living space and residential buildings. The moderate regions were at the outskirts of the study area and more distant to the booming regions (e.g., Nuremberg-Fuerth-Erlangen) and characterized by a decline in residential buildings, and the lowest decline of agricultural land (−0.7%).

**Table 3:** Interpretation of major land-use/land-cover change clusters in the Nuremberg Metropolitan Region (2008–2018)

	Description	Number of municipalities
Cluster 2	Booming regions – Strongest increase in living space and residential buildings – Considerable increase in commercial areas – Considerable loss of agricultural land	90
Cluster 3	Transition regions – Inefficient settlement and traffic infrastructural development – Afforestation hot spots – Strong loss of agricultural land – Decline of the commercial area	39
Cluster 5	Prosperous regions – Considerable increase in living space and residential buildings – Increase in the commercial area	266
Cluster 6	Moderate regions – Lowest increase in settlement area – Decline in residential buildings – Lowest decline of agricultural land	193

### Factors Explaining Cluster Differences

**Fig. 2:**
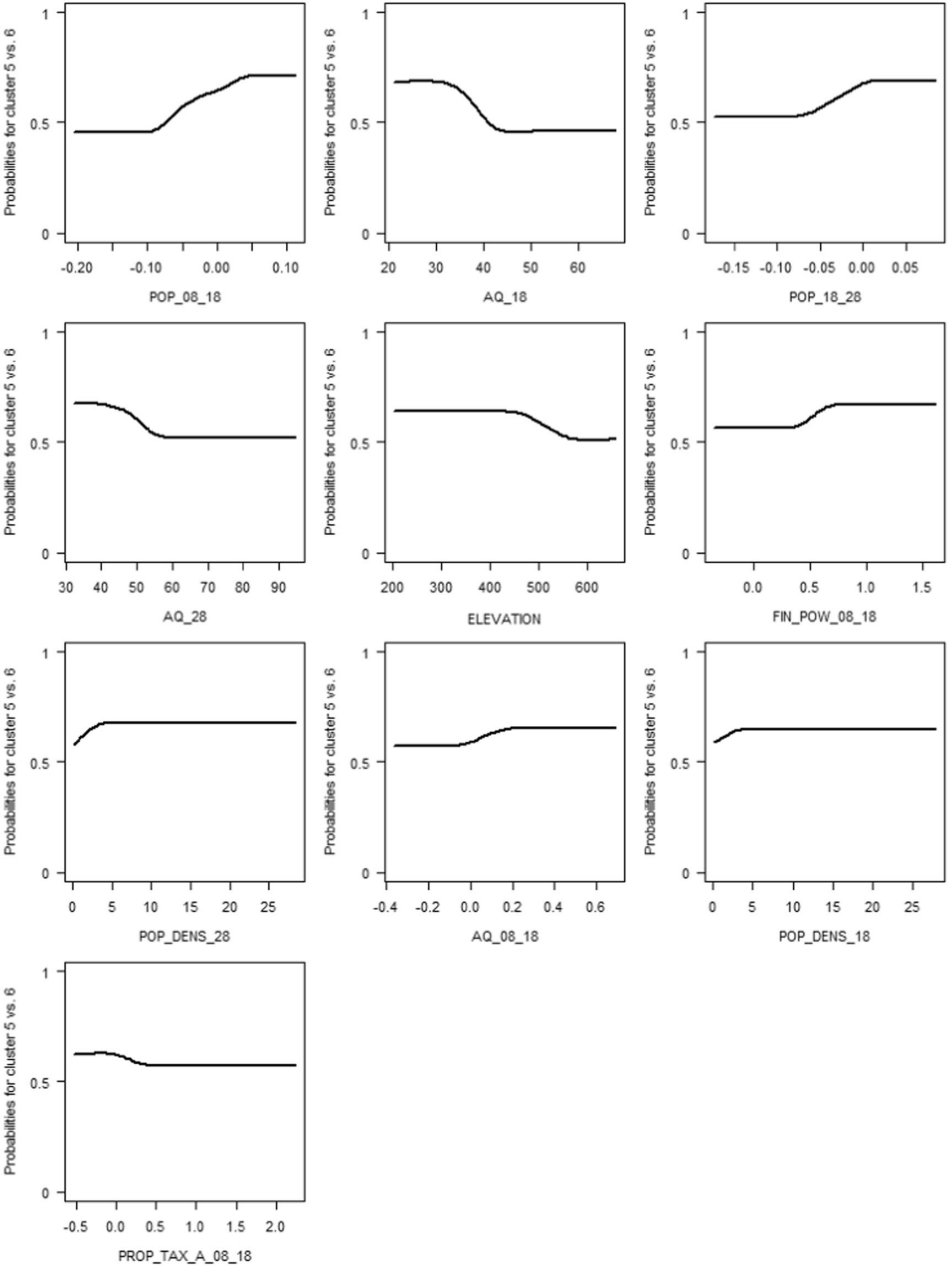
Partial dependency plots for the most influential explanatory variables in descending order of importance, distinguishing prosperous regions (Cluster 5; probability towards 1) from moderate regions (Cluster 6; probability towards 0). Code explanations in brackets: AQ_08_18/AQ_18/AQ_28 (Old-age dependency ratio (2008–2018/2018/2028) [%]), ELEVATION (Elevation [m]), FIN_POW_08_18 (Financial power (2008–2018) [%]), POP_08_18/POP_18_28 (Population development (2008–2018/2018–2028) [%]), POP_DENS_18/POP_DENS_28 (Population density (2018/2028) [n ha^−1^]), PROP_TAX_A_08_18 (Property Tax A (2008–2018) [%])

We analyzed differences between clusters with boosted regression trees. In contrast to moderate regions (cluster 6; probability towards 0), prosperous regions (Cluster 5; probability towards 1) showed a stronger previous and future population growth (2008–2018 and 2018–2028, respectively) (see Fig. 2). Similarly, the current and future old-age dependency ratio (i.e., the number of individuals aged >65 years per 100 people) was lower for prosperous regions. Interestingly, the increase of the old-age dependency ratio from 2008 to 2018 was lower for the moderate regions than for the prosperous regions. Between 2008 and 2018, the financial power increased more strongly in the prosperous regions than in the moderate regions. Regarding major interactions of variables (see Fig. S12 in the supplementary material), prosperous regions had a lower old-age dependency ratio and a higher increase in the population in the future (2018–2028).

In contrast to moderate regions (Cluster 6) (probability towards 0), transition regions (Cluster 3; probability towards 1) (see supplementary material, Fig. S8) had a higher financial power per capita and a population increase (2008 to 2018). Interestingly, the number of jobs and the net commercial tax per capita were lower, and the commuter balance was more likely negative for transition regions. The projected old-age dependency ratio (2028) was equally higher for transition regions. Landscape-wise, transition regions had a negative curvature and a higher slope. Regarding interactions, transition regions have a higher financial power, but more often a lower net commercial tax per capita. The relevant Fig. S13 can be found in the supplementary material.

In contrast to prosperous regions (Cluster 5; probability towards 0), transition regions (Cluster 3; probability towards 1) (see Supplementary material, Fig. S9) had a higher financial power per capita. Contrastingly, tax power and commercial tax power per capita were likely lower in transition regions. Compared with prosperous regions, transition regions also showed a decline or low increase in the number of jobs of the people living in the municipality between 2008 and 2018. Landscape-wise, the slope was higher and the roughness of the municipalities lower. Regarding interactions, a higher financial power per capita despite a low tax power per capita seemed counterintuitive. The relevant Fig. S15 can be found in the supplementary material.

**Fig. 3:**
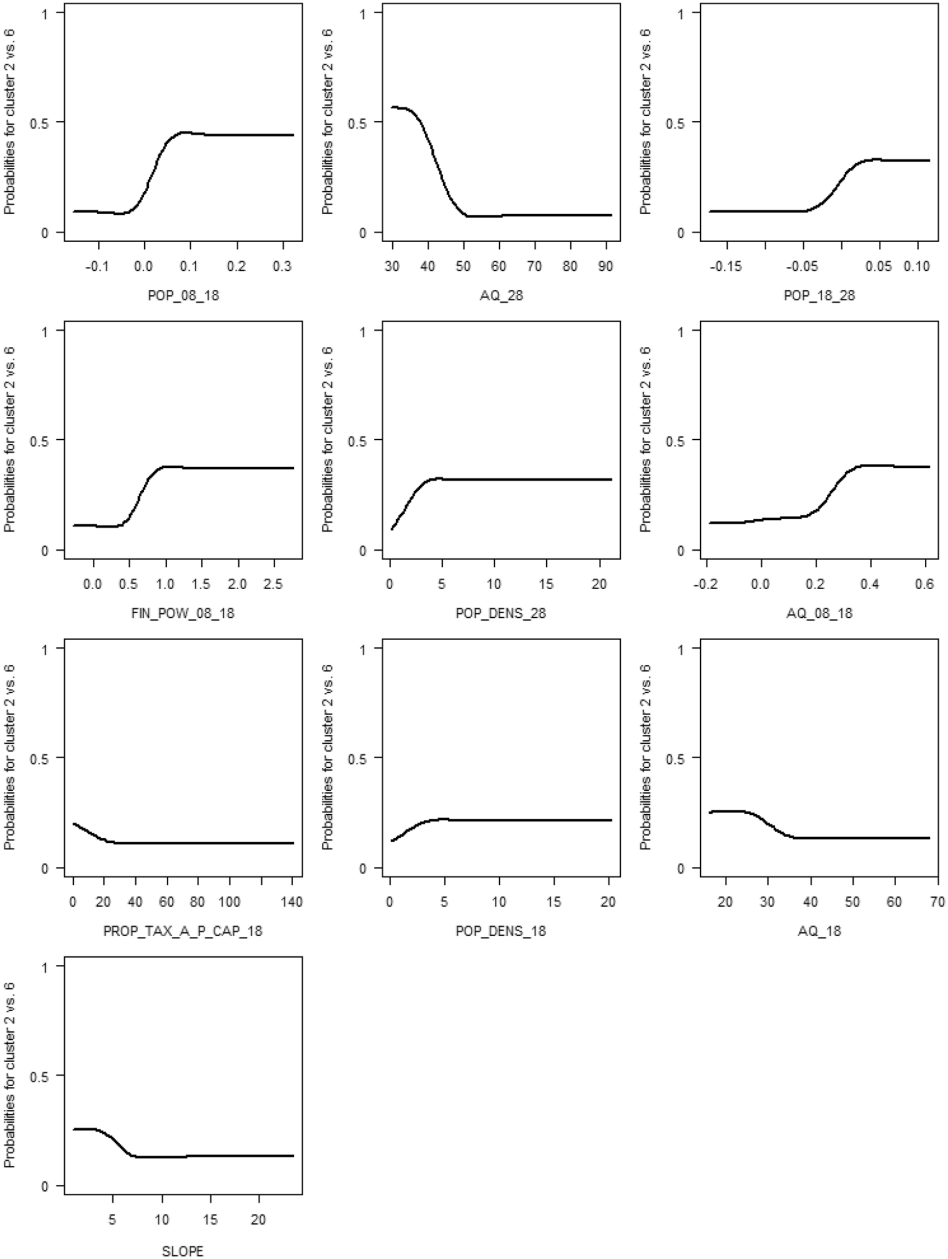
Partial dependency plots for the most influential explanatory variables in descending order of importance, distinguishing booming (Cluster 2; probability towards 1) from moderate regions (Cluster 6; probability towards 0). Code explanations in brackets: AQ_08_18/AQ_18/AQ_28 (Old-age dependency ratio (2008–2018/2018/2028) [%]), FIN_POW_08_18 (Financial power (2008–2018) [%]), POP_08_18/POP_18_28 (Population development (2008–2018/2018–2028) [%]), POP_DENS_18/POP_DENS_28 (Population density (2018/2028) [n ha^−1^]), PROP_TAX_A_P_CAP_18 (Property Tax A per inhabitant (2018) [€ n^−1^]), SLOPE (Slope [%])

In contrast to moderate regions (Cluster 6, probability towards 0), booming regions (Cluster 2, probability towards 0) showed a past and a future increase in population (2008–2028), and a lower old-age-dependency ratio (2028 and 2018) (see Fig. 3). Similarly, the financial power of booming regions increased from 2008 to 2018. Property tax A for non-built-up areas per capita was more likely lower for booming regions than for moderate regions. Regarding major interactions of variables (see Fig. S14 in the supplementary material), a joint increase in population and in the old-age dependency ratio as well as a stronger increase in the financial power (2008–2018) were more likely in booming regions.

In contrast to prosperous regions (Cluster 5; probability towards 0), booming regions (Cluster 2; probability towards 1) showed a past and a future increase in population between 2008 and 2028, and a lower old-age-dependency ratio in 2028 and 2018 (see Supplementary material, Fig. S10). Similarly, the financial power and the number of jobs in booming regions increased more likely between 2008 and 2018. The soil quality rating was either very low or higher in booming regions. Interactions were not analyzed as the correlation coefficient was highest for a tree complexity of 1 (see Supplementary material, Table S6).

In contrast to transition regions (Cluster 3; probability towards 0), booming regions (Cluster 2; probability towards 1) showed a past and future increase in population (2008 to 2028), and in the number of jobs of people living in the municipality (2008 to 2018). Similarly, the financial power of booming regions more likely increased (2008 to 2018), but the financial power per capita was likely lower than for transition regions (2018). Interactions were not analyzed as the correlation coefficient was highest for a tree complexity of 1 (see supplementary material, Table S7).

### Indicators of Land Consumption

We developed an indicator-based monitoring system, which should enable stakeholders, planners, and politicians to assess the current state of individual municipalities and to compare communities with each other. The respective indicators are measures for certain fields of action (e.g., increasing the efficiency of land use, reducing the loss of agricultural land) and condense complex interrelationships into easy-to-use information. In order to compile a comprehensive set of indicators, we conducted an extensive literature analysis of scientific papers and sustainability reports to identify relevant and suitable measures and to define requirements for indicator sets. An overview of the databases and publications evaluated can be found in Table S1 in the supplementary material. Based on guided expert workshops with stakeholders at the regional and local policy level, as well as from land management and planning (e.g., landscape conservation associations, organic agriculture model regions, regional planning associations, and farmers' associations) from spring 2019 until the end of 2020, the project team identified three target dimensions that need to be addressed in holistic land governance at the municipal level: (1) sustainable land use and preservation of sufficient agricultural land for the cultivation of regional products, (2) preservation of the diverse cultural landscapes and their social and ecosystem services, (3) expansion of regional added value and securing markets for typical regional products and specialties. The third target dimension stems from the fact that particularly the valorization and regional supply of typical primary products are intended to trigger sustainable land use and can strengthen the urban-rural relationship within the Metropolitan Region. Subsequently, the collected indicators were evaluated according to the following requirements:

- *Goal-oriented:* indicators provide a clear relation to defined target dimensions and are representative of the geographic region of the Nuremberg Metropolitan Region; involvement of target groups to ensure long-term use is necessary.
- *Sensitive* to measures at the municipal level: decision-makers at the municipal level should be able to influence the target values.
- *Feasible:* indicators have to be comprehensively available at the municipal level and must be regularly updated. To ensure complete temporal and spatial coverage and financial feasibility, the majority of our indicators are based on routinely gathered data sources (e.g., topographic maps or census data). To safeguard long-term feasibility; the number of indicators should remain manageable.
- *Consistent:* indicators should be consistent with recognized concepts in spatial monitoring (Maes et al. 2016; Termorshuizen and Opdam 2009) and provide added value compared to available monitoring programs (e.g., Monitor of Settlement and Open Space Development (IOER Monitor); Indicators and Maps for Space and Urban Development (INKAR); EU SDG Indicator set 2020, European Environment Agency (EEA) indicators)

Advanced and core indicators could be identified, which are particularly suitable for a multidimensional land consumption monitoring scheme of the Nuremberg Metropolitan Region. Core indicators are characterized by the fact that they are valid, that the quality of their data is high, and that the data is readily available throughout the region. If the indicators meet all the requirements but the data is not sufficiently available across the region, they were designated as advanced indicators. The first draft of the indicator set was presented to stakeholders at the conference of the Nuremberg Metropolitan Region in late 2020 and discussed and revised in three further workshops (e.g., with mayors and administrative potential user groups and with land-saving managers). A selection of possible indicators is presented in Table 4.

**Table 4:** Selection of possible indicators for a land-use monitoring system in the Nuremberg Metropolitan Region

Target dimensions/topics	Core and advanced (*italic*) indicators	Effects	Units	Data sources
*Sustainable land use and preservation of sufficient agricultural land for the cultivation of regional products*				
Efficient and effective land use	(Annual) land consumption per inhabitant	Recording the specific adherence to the proportional budget for land consumption (see Leibniz Institute of Ecological Urban and Regional Development (2020) and BBSR (2020))	ha n^−1^	ATKIS^a^, BayLfStat^b^
	Commercial land per employee	Information on the relationship between jobs and the commercial area used and measure of the efficiency of commercial area consumption (see Rusche and Mayr 2011)	ha n^−1^	ATKIS^a^, BayLfStat^b^
	Living space per inhabitant	Information on the relationship between inhabitants and living space used; a measure of efficient consumption of living space (see Leibniz Institute of Ecological Urban and Regional Development (2020) and BBSR (2020))	m² n^−1^	ATKIS^a^, BayLfStat^b^
Ensuring economic profitability	Commercial tax revenue per ha of the commercial area	Information on the ratio of trade tax revenue and the amount of commercial area used; a measure of the efficiency of commercial area consumption with respect to fiscal considerations	€ ha^−1^	ATKIS^a^, BayLfStat^b^
Utilizing land potentials	*Share of land potentials (Vacancy, fallow land, building gap)*	Indicator records possible potentials of inner development through revitalization	%	Not available according to our knowledge; surveys at the municipal level necessary
Reduction of agricultural land losses	Agricultural land per inhabitant/	Recording the potential area for (local) food production with the idea of food security (see Leibniz Institute of Ecological Urban and Regional Development (2020) and BBSR (2020))	ha n^−1^	ATKIS^a^
	Annual conversion of agricultural land	Recording the loss of potential area for (local) food production (Leibniz Institute of Ecological Urban and Regional Development 2020)	%	
Provision of compensation areas	*Share of compensation areas*		%	Ecological Land Registry^c^
*Preservation of the diverse cultural landscapes and their social and ecosystem services*				
Expansion of organic farming	Share of organic farming	Soil conservation and mitigation of harmful pollution of the environment (see Maes et al. (2016), Eurostat (2020) and LIKI (2020))	%	IACS^d^
Grassland conservation	Share of grassland/	Regulating groundwater quality and providing fodder from grasslands (Albert et al. 2016)	%	ATKIS^a^
	Annual conversion of grassland		%	
Regulation services—water, climate, erosion, pollination, pest control	*Percentage of landscape elements of agricultural land*	Mitigation of soil erosion, facilitating pollination and biological pest control (Albert et al. 2016)	%	IACS^d^
	*Crop diversity (Shannon-Index)*	A measure of species richness and of the esthetic quality of the landscape	index	IACS^d^
	*Share of agricultural land under agri-environmental measures*	Identification of environmentally friendly management measures that aim at water, soil and climate protection, biodiversity, and the preservation of cultural landscape	%	IACS^d^
*Expansion of regional added value and securing markets for typical regional products and specialties*				
Maintain regional agricultural production and marketing	*Gross value added per employee in agriculture, forestry, and fisheries*	Indicator provides information on the regional value-added by agricultural production (BBSR (2020))	€ n^−1^	Not available according to our knowledge; surveys at the municipal level necessary
	*Number of farms with direct marketing per 1000 inhabitants*	Regionally produced products sold through direct marketing lead to a higher added value for the farmer	n 1000 n^−1^	Not available according to our knowledge; surveys at the municipal level necessary
Maintain employment	Employees in agriculture, forestry, fisheries per agricultural area	Information on the labor intensity of agricultural production (BBSR (2020))	n ha^−1^	BayLfStat^b^

^a^ATKIS authoritative topographic-cartographic information system

^b^GENESIS online database, the Bavarian state office for statistics

^c^Ecological land registry, Bavarian environment agency

^d^IACS integrated administration and control system, the Bavarian Ministry for food, agriculture, and forestry

## Discussion

### Clusters of Land-Use/Land-Cover Change

The results showed four major clusters that describe land-use/land-cover change in the Nuremberg Metropolitan Region with an emphasis on settlement and infrastructural landscape dynamics. They go beyond existing studies that focus on explaining land-use/land-cover change at the local level (e.g., Nuissl et al. 2009 and Salvati et al. 2018) or at the European level in a comparative manner (e.g., Salvati et al. 2018 and Levers et al. 2018a). However, the regional and subnational scale is mostly understudied and covered by this study. Because of a strong path dependency in land-use/land-cover change and heterogenous land-use/land-cover patterns, the resulting clusters from this study cannot be directly transferred to other regions. Nevertheless, we observe trajectories of land-use/land-cover change in our study area, which might be transferable to other European regions. In the context of European patterns of land consumption as identified by Kuemmerle et al. (2016), the Nuremberg Metropolitan Region is an example for widely “spread” land consumption without very pronounced centers of increasing land consumption. Regions with comparable patterns can, for example, be found in Central and Northern Germany, parts of the Czech Republic, or Ireland (outside urban agglomerations). This case study may contrast highly urbanizing areas in the Netherlands or Belgium, and in coastal or inland agglomerations in France and Southwestern Europe. The conversion of agricultural land-use /land-cover change in the Nuremberg Metropolitan Region is mostly representative for slightly mountainous areas in Austria and Germany given comparable structures of the land system (Meyer and Früh-Müller 2020).

The used approach of self-organizing maps identified and revealed clear patterns of land-use/land-cover change. The approach has been used for clustering trajectories and patterns in land systems across Europe, where local or regional patterns may not be well-revealed due to the heterogeneity of landscape in the European context (Levers et al. 2018a). Besides, this study focuses on settlement and infrastructure-driven land-use/land-cover change patterns and likely provides different land-use/land-cover change clusters when all land-cover/land-use classes are equally considered.

### Factors Explaining Cluster Differences

We analyzed the relationships between land consumption and potential socio-demographic, economic, and environmental drivers. We used boosted regression trees in order to be able to identify non-linear relationships between land consumption and explanatory variables as shown for drivers of agricultural land-use/land-cover change (Meyer and Früh-Müller 2020). Existing studies that analyze the impact of socio-demographic factors on land-use/land-cover change patterns mostly use linear regression techniques (e.g., for the explanation of surface sealing Behnisch et al. 2016). Other studies (Dittrich et al. 2017; Levers et al. 2018a) test the correlation or spatial overlap between clusters of land-use/land-cover change or ecosystem services with socio-demographic clusters of multiple variables based on self-organizing maps but do not focus on the relevance of individual and potential drivers of land-use/land-cover change.

This study showed that demographic trends usually considered favorable (population growth and lower old-age dependency ratio) seem to come at the expense of considerable settlement and traffic infrastructure development. This pattern is reflected in increased living space and commercial area development as well as in the considerable number of jobs (see the comparisons between Cluster 2 (booming regions), Cluster 3 (transition region), and Cluster 5 (prosperous regions) in the results section). This study thereby partly confirms Kroll and Haase (2010). In moderate regions, population decline is associated with moderate land consumption, whereas the economic growth and fiscal stability of municipalities seemed to be bound to land consumption. This is also reflected in the comparison of booming regions (Cluster 2) with other regions/clusters in this study. Although large parts of the study area were dominated by land consumption and fiscal prosperity, booming regions seemed to be more efficient in settlement development (lower space consumption for additional buildings for living despite a population increase).

Cluster 3 (transition regions) showed a very interesting pattern, which is likely driven by and subject to a slight population decline. Transition regions seemingly aim to attract population through considerable living space development but do not seem to strongly counteract a decline in agricultural land and a stagnation of commercial areas. This effect seems to be supported by a high financial power per capita compared to other clusters. Such a cluster reflects a general trend of an increasing commuting distance between the place of work and residence (Galvin and Madlener 2014), which could be named “dormitory and leisure regions”. This strategy is partly linked to certain economic factors for the industrial and service sectors (e.g., manifested in a decline in commercial areas and a weak commercial tax) and environmental conditions (e.g., high afforestation rates and a decline of agricultural land due to low soil quality and relatively strong relief as shown by Meyer and Früh-Müller 2020), which are favorable for recreation and less favorable for agriculture (Paracchini et al. 2014).

However, it is unclear how a potential increase in remote working habits (e.g., through changed working modes spurred through COVID-19) might affect the distance between the place of work and residence. Germany has a considerable backlog with a below-average remote working rate of 11% (2017) compared to other European countries with rates above 30% such as the Netherlands, Luxembourg, or Sweden (Crößmann et al. 2018). It would be interesting to study how the current pattern of considerable living space and commercial area development in booming regions will dilute to other regions. The composition of private and commercial land consumption could be equally affected (e.g., declining office space vs. increasing demand for living space due to increased time at home). In that respect, it would be fruitful to study for different scenarios, how land consumption is affected in different regions and which factors govern the potential dynamics (e.g., will locational factors that drive land consumption change from economic to leisure factors?).

Two pathways of regional development and land consumption became apparent: increasing, but inefficient land consumption for prosperity (e.g., in transition and moderate regions) and more efficient land consumption in prosperous and booming regions. These patterns partly agree with Mascarenhas et al. (2019), who identified a “dumb decline” characterized by inefficient settlement development pathways in less prosperous regions in Portugal. The role of land markets seems to be as following: difficult current or future economic and/or demographic situations of municipalities seem to foster inefficient settlement development by lowering the prices for land or at least by offering land at lower prices (see, e.g., Henger and Bizer 2010). This market situation governed by local policymakers leads to higher land consumption per capita for living space and commercial areas. The latter is independent of land consumption for infrastructure, which is typically higher for less densely populated areas, which are mostly the transition regions and, to a limited extent, the prosperous and moderate regions. Therefore, inefficiencies are an effect of consumption and housing preferences (e.g., detached housing, easily accessible local suppliers) and business strategies in the non-booming regions to attract new inhabitants or industries.

### Implications for Governance

Existing legislation in Germany hardly sets strict rules for land consumption for decision-makers at the municipal level. Municipal land-use planning sovereignty is guaranteed by the federal constitution (Köck and Bovet 2008). Therefore, major governance mechanisms such as land trading permits address the “land sovereign”, i.e., the municipalities (e.g., Meub et al. 2017). This sovereignty allows most municipalities to increase residential and commercial areas, which are their major (perceived) means to improve their financial strength as most municipal income comes from commercial tax, income tax, or allowances for inhabitants (Bizer 2006). This favors increasing commercial activities and the designation of settlement development areas to attract new inhabitants at the expense of neighboring municipalities or long-term fiscal stability of the own municipality (Fischer et al. 2009; Meub et al. 2017). In Germany, there is not a single system for the allocation or distribution of permits for land consumption in place beyond a pilot stage (Meub et al. 2017). Current governance effects on land consumption and soil sealing are informal instruments (e.g., information for municipalities, practitioners, or citizens) as shown for Munich and Leipzig (Artmann 2014), but which are often the least efficient compared with the regulation or financial incentives (Kment 2018). It has been shown that a mix of legal planning instruments and economic taxes could be most efficient if adequately applied (Nuissl and Schroeter-Schlaack 2009). The inadequate mix of governance instruments is reflected in the inefficiencies in land consumption in our study region.

The indicator-based land-use monitoring system described here is an attempt to initiate awareness of the impacts of land consumption on ecosystem goods and services, regional (food) supply, and sustainable economic development towards inclusive landscape governance. It aims at moving beyond administrative silos (see, e.g., Ros-Tonen et al. 2018). The monitoring system has been exemplarily developed for the municipal level within the Nuremberg Metropolitan Region. Even though the structure of clusters and related drivers will highly likely vary, the monitoring system can be easily transferred to other regions due to its flexibility in indicators used. Those indicators will be operationalized and entered into a user-friendly Web-GIS interface to enable stakeholders, planners, and policymakers to easily access information. The provision of reliable datasets on the current state of land management and observed developments of land-use/land-cover change effects can improve inclusive decision-making at the local level and hopefully mitigate land consumption in the long run. However, the practicability of the indicator-based monitoring has to be evaluated in the daily routine of the planning processes. The indicator set has been intensively discussed with local practitioners, evaluated, and adjusted in the subsequent implementation phase of the project (see https://reprola.de/). The selection of indicators based on literature analysis and expert workshops prioritizes those indicators that can capture many aspects of land consumption and ensure long-term maintenance at the same time (feasibility in data maintenance, costs, expertise, etc.). Due to the secondary focus on regional agricultural added value, we see aspects to be more thoroughly covered in other indicator sets. These include the resilience of land use, landscape aesthetics and recreation, regional energy production, and other aspects of economic competitiveness besides the agricultural sector (e.g., Hersperger et al. 2017).

### Limitations

We analyzed multiple demographic, economic, infrastructural, and biophysical factors, which could affect land consumption. If possible, we tested the explanatory power of static (current values) and dynamic indicators (temporal changes of variables if available). However, some variables such as internet access or public transport infrastructure were not available for the starting and the end year of the study.

Data on land-use/land-cover was not consistently available for the given time period due to changes in land surveying in Bavaria. Bavaria changed the surveying method to quantify actual land cover instead of property boundaries, which mostly limited one property to one land-cover class. However, data following the old and new methodology was available for three consecutive years starting in 2011 (Penn-Bessel 2018). Therefore, we added the relative changes in land-use/land-cover change for the years with the same methodology prior to and past the methodological change to overcome the data distortion. For this reason, we were only able to quantify relative, but no nominal changes for the analyzed period, but could analyze land-use/land-cover change patterns in the recent decade. In the future, it would be relevant to test whether the identified patterns in this study also hold for datasets without the mentioned distortions in land surveying.

Due to changes in land surveying, we had to exclude landscape structure as a characteristic of land consumption. The distortion between different years was too high to allow for a comparison of landscape structural changes at the municipal level. However, future studies without the given distortions in data should include landscape structure as a potential driver for land consumption. Cluster 3 (transition regions), which is characterized by fragmented agricultural landscapes, seems to be highly susceptible to conversion towards forests compared to regions with considerably larger plots. Considering landscape structure could improve the study (Meyer and Früh-Müller 2020).

## Conclusions

Municipalities with more efficient settlement development are often booming regions with scarce and more expensive land. In contrast, economically weaker municipalities with a declining population or less favorable demographics and likely lower land prices are often less efficient in settlement development. This inefficiency in settlement development has a clear effect on land market prices and on land consumption. This trend towards offering more land for settlement was apparent in demographically and economically weaker regions. In that respect, governance mechanisms such as regional planning or subsidy schemes for rural development are currently unable to counteract these environmentally and fiscally inefficient land consumption patterns. Major actors in land management (especially municipal policymakers) put the rational first: economic development potential (e.g., through commercial area development) and population growth (e.g., through new settlement development areas). Major actors’ decision-making is guided by insufficient quantitative information on trade-offs between economic, social, and environmental dimensions.

Technically, knowledge on sound indicator sets on land consumption (c.f. Siedentop and Fina (2010) or Marquard et al. (2020) for an overview) and its impacts (e.g., for decision support systems in spatial planning) exists (Grêt-Regamey et al. 2017b). Our indicator-based monitoring system aggregates information at the municipal level as the main decision-level of urban-land-use planning in Germany. With an intermediate aggregation level, this is an addition to existing studies and monitoring tools that are based on coarser scales beyond municipalities (e.g., Hersperger et al. (2017)) and to studies using finer scales that assess the impact of land consumption and urban land-use planning (Grêt-Regamey et al. 2017a).

The monitoring system aims to be informative for municipal decision-makers to support and improve their land management. The proposed monitoring tool aims at broadening the perspective from purely economic or demographic narratives towards a holistic perspective on the impacts of settlement development. Here, the main contribution is to broaden the perspective to fiscal stability, economic prosperity, quality of life, or environmental health in an integrative manner. In addition, the coherent and independent assessment across municipalities puts the municipal land-use planning impacts into perspective, providing a comparison with neighboring communities. The possibility of an inter-municipal benchmarking may help to overcome the trap of the inter-municipal competition with land for inhabitants and companies.

Further research is needed to test how this intermediate approach at the municipal level impacts actual decision-making. It should assess whether tools such as the proposed indicator framework is able to sufficiently or considerably reduce land consumption and to spatially allocate land for settlement development with higher efficiency in terms of ecosystem services or other benefits. In addition, the limitations of informational governance should be further elaborated given the known need for a mix of governance instruments.

## Supplementary information

Supplementary Information

## Footnotes

1 Surface sealing is understood as the process of covering soils with impermeable materials such as asphalt and concrete (European Commission 2021).
