# Supplementary material for: Spatial Indicators to Monitor Land Consumption for local Governance in Southern Germany
Source: Environ Manage. 2021 Mar 22;68(5):755–71. doi: 10.1007/s00267-021-01460-3 (PMC7983356; doi:10.1007/s00267-021-01460-3)
Supplement: Supplementary file 1 — Supplementary Information [file 267_2021_1460_MOESM1_ESM.docx]

**Table S1: Selection of the evaluated publications and monitoring schemes in order to compile a comprehensive and suitable set of indicators which enables stakeholders to better govern land use at the level of municipalities.**

| Monitoring schemes | - Indicators for mapping the United Nations Sustainable Development Goals in German municipalities (Assmann, Honold, Grabow, & Roose, 2018) - Indicators and maps of spatial and urban development (BBSR, 2019) - Sustainable Municipality Monitor (Riedel, et al., 2016) - [Monitor of Settlement and Open Space Development (IOER Monitor)](https://www.ioer-monitor.de/en/) (Leibnitz-Institut für Ökologische Raumentwicklung, 2020) - Federal State Initiative – Core Indicators – LIKI (LIKI, 2021) - Indicators for the assessment of sustainable and climate change compatible settlement development in (Behnisch, Kretschmer, & Meinel, 2018) |
| --- | --- |
| Scientific publications | - Indicators and reference values for Swiss landscapes (Hersperger, Mueller, Knöpfel, Siegfried, & Kienast, 2017) - nationwide indicators for the assessment of ecosystem services (Albert, et al., 2015) - indicator framework for assessing ecosystem services in support of the EU Biodiversity Strategy to 2020 (Maes, et al., 2015) |

References:

Assmann, Dirk, Jasmin Honold, Busso Grabow und Jochen Roose. SDG-Indikatoren für Kommunen – Indikatoren zur Abbildung der Sustainable Development Goals der Vereinten Nationen in deutschen Kommunen. Hrsg. Bertelsmann Stiftung, Bundesinstitut für Bau-, Stadt- und Raumforschung, Deutscher Landkreistag, Deutscher Städtetag, Deutscher Städte- und Gemeindebund, Deutsches Institut für Urbanistik, Engagement Global. Gütersloh 2018.

BBSR. INKAR - Indikatoren und Karten zur Raum- und Stadtentwicklung. 2019. http://dx.doi.org/10.1007/978-3-662-50305-8. (Accessed March 31st, 2020).

Riedel, H., et al. Monitor Nachhaltige Kommune. Bericht 2016 - Teil 1. Ergebnise der Befragung und der Indikatorenentwicklung. Gütersloh: Bertelsmann Stiftung, Deutsches Institut für Urbanistik., 2016.

Leibnitz-Institut für Ökologische Raumentwicklung. Monitor der Siedlungs- und Freiraumentwicklung - IÖR-Monitor. 2020. https://www.ioer-monitor.de/ (Accessed March 27th, 2020).

LIKI. Länderinitiative Kernindikatoren. 2021. https://www.lanuv.nrw.de/liki/index.php?mode=home (Accessed January 19th, 2021).

Behnisch, Martin, Odette Kretschmer, und Gotthard Meinel. Flächeninanspruchnahme in Deutschland. Auf dem Wege zu einem besseren Verständnis der Siedlungs- und Verkehrsflächenentwicklung. Berlin, Heidelberg: Springer Spektrum, 2018.

Hersperger, A. M., G. Mueller, M. Knöpfel, Angelika Siegfried, und F. Kienast. „valuating outcomes in planning. Indicators and reference values for Swiss landscapes.“ Ecological Indicators, 2017.

Albert, Christian, Benjamin Burkhard, Sabrina Daube, Katharina Dietrich, Barbara Engels, und Jakob et al. Frommer. Empfehlungen zur Entwicklung bundesweiter Indikatoren zur Erfassung von Ökosystemleistungen. Bde. BfN-Skripten, 410. Bonn: BfN, 2015.

Maes, J., et al. „An indicator framework for assessing ecosystem services in support of the EU Biodiversity Strategy to 2020.“ Ecosystem Services, 22. 11 2015: 14-23.

Identifying relevant clusters of land-use/land-cover change


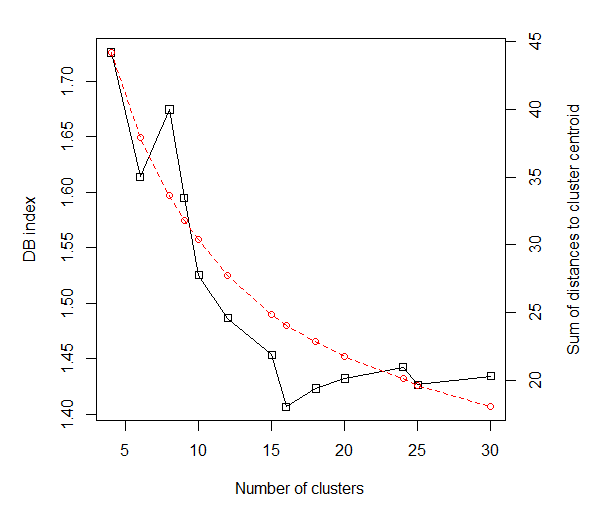

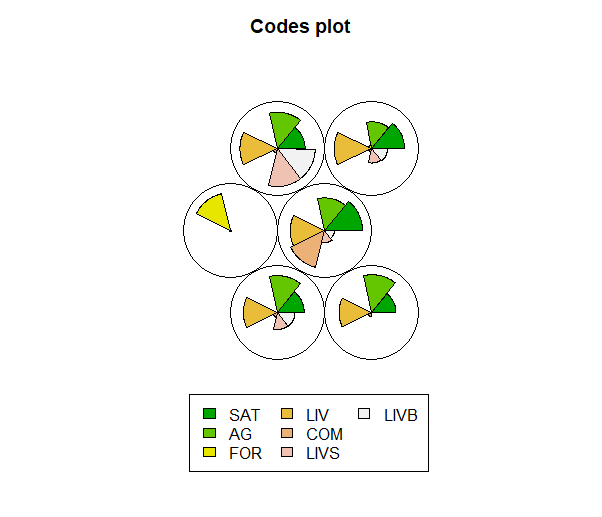


**Fig. S1: Identifying the optimal number of clusters for land-use/land-cover change trajectories (left) and visualization of clusters and their proximity (right); a low Davies-Bouldin (DB) index (squares) shows a high inter-cluster and low intra-cluster variability. The sum of the Euclidean distance to the respective cluster centroid of all clusters indicates through its changes in slope whether to decide for a reduced number of clusters vs. distinct cluster characteristics. We chose a 2x3- hexagonal plane with six clusters.**

Additional figures supporting the analysis of cluster differences with boosted regression trees

**Table S2: Calibration of the boosted regression tree for the learning rate and tree complexity for distinguishing prosperous (cluster 5) from moderate regions (cluster 6); the performance was evaluated through 10-fold cross-validated correlation coefficients; we highlighted the chosen combination in bold.**

|  | | **Learning rate** | | | | | | | | **row mean** |
| --- | --- | --- | --- | --- | --- | --- | --- | --- | --- | --- |
|  |  | **0.00025** | **0.0005** | **0.00075** | **0.001** | **0.0025** | **0.005** | **0.0075** | **0.01** |  |
| **Tree complexity** | ***1*** | 0.516 | 0.523 | 0.516 | 0.523 | 0.504 | 0.508 | 0.505 | 0.507 | 0.513 |
|  | ***2*** | 0.512 | 0.527 | 0.532 | 0.515 | 0.517 | 0.510 | 0.518 | 0.517 | 0.519 |
|  | ***3*** | 0.519 | 0.527 | 0.519 | 0.519 | 0.515 | 0.519 | 0.517 | 0.513 | 0.519 |
|  | ***4*** | 0.516 | 0.525 | 0.530 | 0.540 | 0.527 | 0.507 | 0.530 | 0.520 | 0.524 |
|  | ***5*** | 0.521 | 0.519 | 0.514 | 0.539 | 0.520 | 0.513 | 0.524 | 0.524 | 0.522 |
|  | ***6*** | 0.526 | 0.531 | 0.532 | 0.512 | 0.518 | 0.534 | 0.528 | 0.530 | 0.526 |
|  | ***7*** | 0.527 | 0.522 | 0.539 | 0.528 | 0.525 | 0.523 | 0.532 | 0.519 | 0.527 |
|  | ***8*** | 0.535 | **0.543** | 0.525 | 0.525 | 0.525 | 0.532 | 0.526 | 0.533 | **0.531** |
|  | ***9*** | 0.537 | 0.534 | 0.530 | 0.507 | 0.530 | 0.536 | 0.523 | 0.534 | 0.529 |
| **column mean** | | 0.523 | **0.528** | 0.526 | 0.523 | 0.520 | 0.520 | 0.523 | 0.522 |  |

**
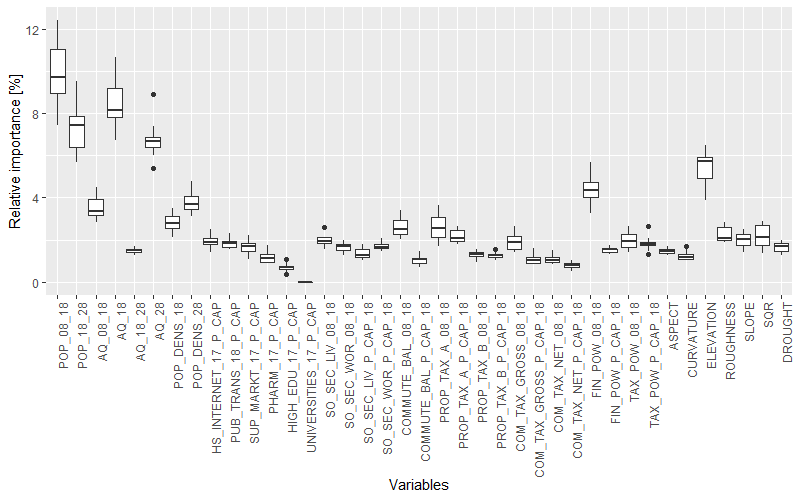
**

**Fig. S2: Relative importance of explanatory variables distinguishing prosperous (cluster 5) from moderate regions (cluster 6) (10 folds each); explained variance: 54.47%.**

**Table S3: Calibration of the boosted regression tree for the learning rate and tree complexity for distinguishing transition (cluster 3) from moderate regions (cluster 6); the performance was evaluated through 10-fold cross-validated correlation coefficients; we highlighted the chosen combination in bold.**

|  | | **Learning rate** | | | | | | | | **row mean** |
| --- | --- | --- | --- | --- | --- | --- | --- | --- | --- | --- |
|  |  | **0.00025** | **0.0005** | **0.00075** | **0.001** | **0.0025** | **0.005** | **0.0075** | **0.01** |  |
| **Tree complexity** | ***1*** | 0.442 | 0.401 | 0.464 | 0.434 | 0.417 | 0.420 | 0.472 | 0.382 | 0.429 |
|  | ***2*** | **0.461** | 0.470 | 0.458 | 0.462 | 0.462 | 0.469 | 0.466 | 0.465 | **0.464** |
|  | ***3*** | 0.475 | 0.433 | 0.446 | 0.415 | 0.435 | 0.481 | 0.453 | 0.490 | 0.453 |
|  | ***4*** | 0.454 | 0.376 | 0.434 | 0.463 | 0.426 | 0.474 | 0.407 | 0.455 | 0.436 |
|  | ***5*** | 0.425 | 0.381 | 0.433 | 0.484 | 0.463 | 0.438 | 0.460 | 0.494 | 0.447 |
|  | ***6*** | 0.469 | 0.454 | 0.433 | 0.453 | 0.433 | 0.454 | 0.453 | 0.499 | 0.456 |
|  | ***7*** | 0.472 | 0.421 | 0.442 | 0.461 | 0.455 | 0.428 | 0.457 | 0.471 | 0.451 |
|  | ***8*** | 0.482 | 0.425 | 0.448 | 0.420 | 0.439 | 0.456 | 0.444 | 0.435 | 0.444 |
|  | ***9*** | 0.444 | 0.391 | 0.433 | 0.416 | 0.481 | 0.434 | 0.398 | 0.418 | 0.427 |
| **column mean** | | **0.458** | 0.417 | 0.443 | 0.445 | 0.446 | 0.450 | 0.446 | 0.456 |  |

**
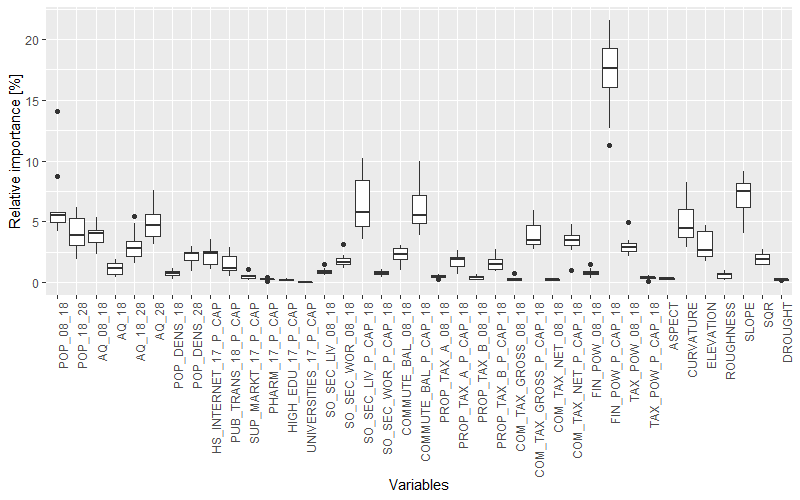
**

**Fig. S3: Relative importance of explanatory variables distinguishing transition (cluster 3) from moderate regions (cluster 6) (10 folds each); explained variance: 46.38%.**

**Table S4: Calibration of the boosted regression tree for the learning rate and tree complexity for distinguishing booming (cluster 2) from moderate regions (cluster 6); the performance was evaluated through 10-fold cross-validated correlation coefficients; we highlighted the chosen combination in bold.**

|  | | **Learning rate** | | | | | | | | **row mean** |
| --- | --- | --- | --- | --- | --- | --- | --- | --- | --- | --- |
|  |  | **0.00025** | **0.0005** | **0.00075** | **0.001** | **0.0025** | **0.005** | **0.0075** | **0.01** |  |
| **Tree complexity** | ***1*** | 0.793 | 0.824 | 0.837 | 0.817 | 0.835 | 0.829 | 0.842 | 0.842 | 0.827 |
|  | ***2*** | 0.808 | 0.822 | 0.826 | 0.832 | 0.823 | 0.833 | 0.827 | 0.833 | 0.826 |
|  | ***3*** | 0.823 | 0.824 | 0.828 | 0.836 | 0.831 | 0.821 | 0.833 | 0.827 | 0.828 |
|  | ***4*** | 0.827 | 0.823 | 0.814 | 0.836 | 0.836 | 0.828 | 0.847 | 0.827 | 0.830 |
|  | ***5*** | 0.818 | 0.825 | 0.817 | 0.811 | 0.830 | 0.828 | 0.843 | 0.832 | 0.825 |
|  | ***6*** | 0.827 | 0.832 | 0.822 | 0.839 | 0.823 | 0.833 | **0.841** | 0.836 | **0.832** |
|  | ***7*** | 0.820 | 0.818 | 0.824 | 0.825 | 0.843 | 0.815 | 0.833 | 0.822 | 0.825 |
|  | ***8*** | 0.817 | 0.835 | 0.825 | 0.820 | 0.831 | 0.813 | 0.821 | 0.833 | 0.824 |
|  | ***9*** | 0.824 | 0.825 | 0.825 | 0.834 | 0.821 | 0.833 | 0.842 | 0.815 | 0.827 |
| **column mean** | | 0.818 | 0.825 | 0.824 | 0.828 | 0.830 | 0.8258 | **0.837** | 0.830 |  |

**
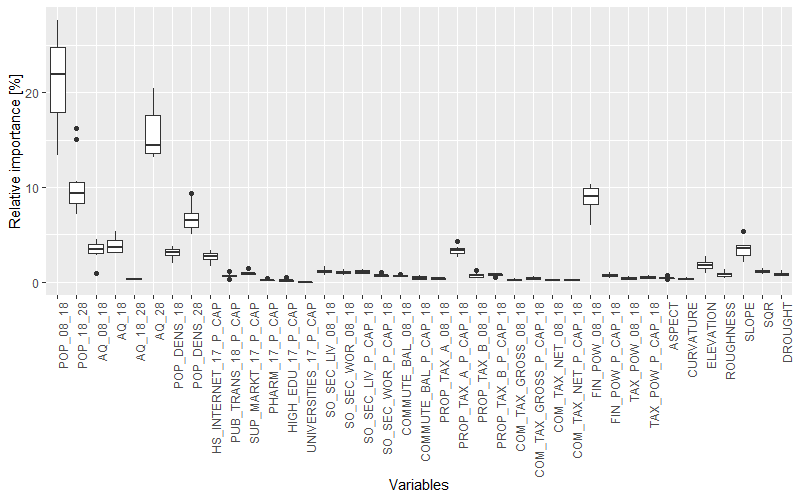
**

**Fig. S4: Relative importance of explanatory variables distinguishing booming (cluster 2) from moderate regions (cluster 6) (10 folds each), explained variance: 90.36 %.**

**Table S5: Calibration of the boosted regression tree for the learning rate and tree complexity for distinguishing transition (cluster 3) from prosperous regions (cluster 5); the performance was evaluated through 10-fold cross-validated correlation coefficients; we highlighted the chosen combination in bold.**

|  | | **Learning rate** | | | | | | | | **row mean** |
| --- | --- | --- | --- | --- | --- | --- | --- | --- | --- | --- |
|  |  | **0.00025** | **0.0005** | **0.00075** | **0.001** | **0.0025** | **0.005** | **0.0075** | **0.01** |  |
| **Tree complexity** | ***1*** | 0.403 | 0.485 | 0.489 | 0.429 | 0.443 | 0.461 | 0.496 | 0.504 | 0.464 |
|  | ***2*** | 0.536 | 0.539 | 0.510 | 0.462 | 0.474 | 0.495 | 0.491 | 0.550 | 0.507 |
|  | ***3*** | 0.493 | 0.445 | 0.516 | 0.508 | 0.525 | 0.502 | 0.446 | 0.458 | 0.487 |
|  | ***4*** | 0.507 | 0.499 | 0.452 | 0.533 | 0.495 | 0.500 | 0.502 | 0.506 | 0.499 |
|  | ***5*** | 0.497 | 0.519 | **0.546** | 0.554 | 0.512 | 0.505 | 0.507 | 0.538 | **0.522** |
|  | ***6*** | 0.554 | 0.508 | 0.479 | 0.523 | 0.475 | 0.506 | 0.530 | 0.534 | 0.514 |
|  | ***7*** | 0.495 | 0.527 | 0.513 | 0.550 | 0.539 | 0.503 | 0.484 | 0.503 | 0.514 |
|  | ***8*** | 0.537 | 0.460 | 0.553 | 0.532 | 0.495 | 0.490 | 0.483 | 0.495 | 0.506 |
|  | ***9*** | 0.489 | 0.508 | 0.548 | 0.479 | 0.536 | 0.499 | 0.503 | 0.512 | 0.509 |
| **column mean** | | 0.501 | 0.499 | **0.512** | 0.508 | 0.499 | 0.496 | 0.494 | 0.511 |  |

**
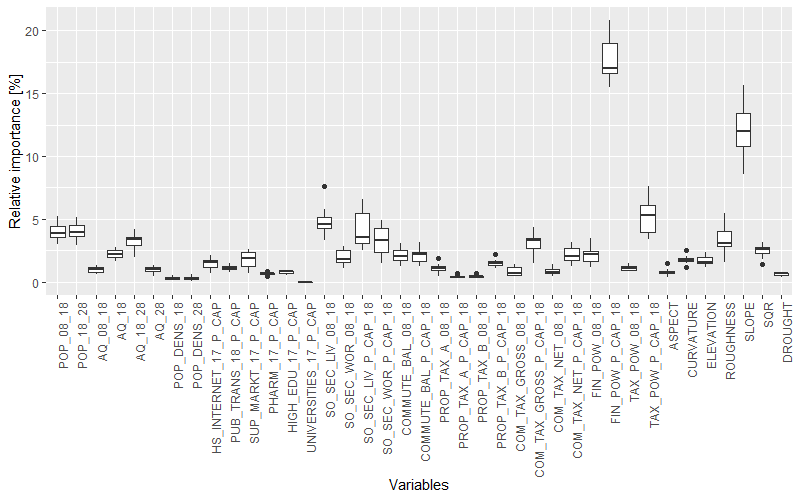
**

**Fig. S5: Relative importance of explanatory variables distinguishing transition (cluster 3) from prosperous regions (cluster 5) (10 folds each); explained variance: 65.32%.**

**Table S6: Calibration of the boosted regression tree for the learning rate and tree complexity for distinguishing booming (cluster 2) from prosperous regions (cluster 5); the performance was evaluated through 10-fold cross-validated correlation coefficients; we highlighted the chosen combination in bold.**

|  | | **Learning rate** | | | | | | | | **row mean** |
| --- | --- | --- | --- | --- | --- | --- | --- | --- | --- | --- |
|  |  | **0.00025** | **0.0005** | **0.00075** | **0.001** | **0.0025** | **0.005** | **0.0075** | **0.01** |  |
| **Tree complexity** | ***1*** | 0.479 | 0.488 | 0.486 | 0.493 | 0.487 | **0.493** | 0.474 | 0.504 | **0.488** |
|  | ***2*** | 0.470 | 0.476 | 0.467 | 0.483 | 0.469 | 0.475 | 0.488 | 0.499 | 0.478 |
|  | ***3*** | 0.464 | 0.492 | 0.473 | 0.460 | 0.464 | 0.495 | 0.464 | 0.480 | 0.474 |
|  | ***4*** | 0.492 | 0.450 | 0.485 | 0.491 | 0.459 | 0.492 | 0.486 | 0.481 | 0.479 |
|  | ***5*** | 0.471 | 0.465 | 0.480 | 0.457 | 0.474 | 0.472 | 0.486 | 0.480 | 0.473 |
|  | ***6*** | 0.486 | 0.498 | 0.497 | 0.464 | 0.472 | 0.498 | 0.473 | 0.467 | 0.482 |
|  | ***7*** | 0.468 | 0.486 | 0.478 | 0.500 | 0.485 | 0.476 | 0.468 | 0.467 | 0.478 |
|  | ***8*** | 0.480 | 0.486 | 0.473 | 0.489 | 0.482 | 0.470 | 0.490 | 0.454 | 0.478 |
|  | ***9*** | 0.469 | 0.480 | 0.470 | 0.476 | 0.469 | 0.471 | 0.463 | 0.464 | 0.470 |
| **column mean** | | 0.475 | 0.480 | 0.479 | 0.479 | 0.473 | **0.483** | 0.477 | 0.477 |  |

**
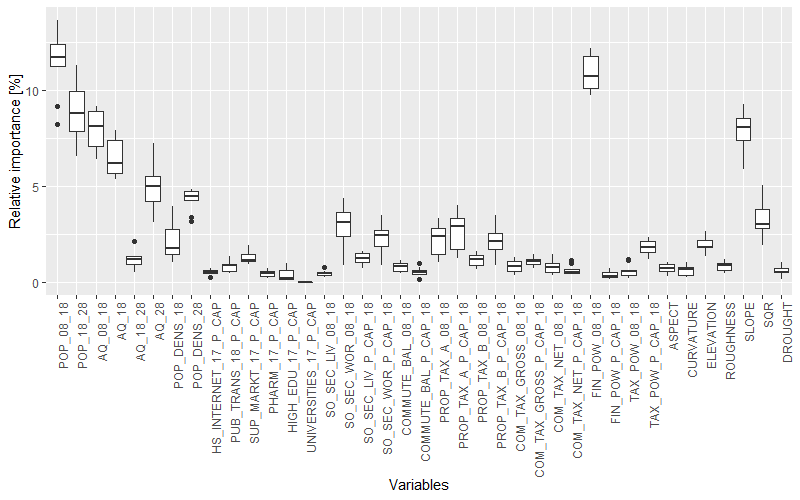
**

**Fig. S6: Relative importance of explanatory variables distinguishing booming (cluster 2) from prosperous regions (cluster 5) (10 folds each); explained variance: 42.43%.**

**Table S7: Calibration of the boosted regression tree for the learning rate and tree complexity for distinguishing booming (cluster 2) from transition regions (cluster 3); the performance was evaluated through 10-fold cross-validated correlation coefficients; we highlighted the chosen combination in bold.**

|  | |  | **Learning rate** | | | | | | | | | **row mean** |
| --- | --- | --- | --- | --- | --- | --- | --- | --- | --- | --- | --- | --- |
|  |  | **0.00025** | | **0.0005** | **0.00075** | **0.001** | **0.0025** | **0.005** | **0.0075** | **0.01** | **0.025** |  |
| **Tree complexity** | ***1*** | 0.743 | | 0.783 | 0.802 | **0.801** | 0.816 | 0.830 | 0.826 | 0.844 | 0.805 | **0.805** |
|  | ***2*** | 0.752 | | 0.811 | 0.795 | 0.821 | 0.800 | 0.798 | 0.805 | 0.798 | 0.797 | 0.797 |
|  | ***3*** | 0.769 | | 0.798 | 0.797 | 0.812 | 0.805 | 0.807 | 0.799 | 0.788 | 0.797 | 0.797 |
|  | ***4*** | 0.794 | | 0.809 | 0.770 | 0.828 | 0.762 | 0.790 | 0.762 | 0.784 | 0.787 | 0.787 |
|  | ***5*** | 0.738 | | 0.788 | 0.787 | 0.797 | 0.785 | 0.802 | 0.800 | 0.764 | 0.783 | 0.783 |
|  | ***6*** | 0.756 | | 0.800 | 0.799 | 0.803 | 0.786 | 0.803 | 0.784 | 0.780 | 0.789 | 0.789 |
|  | ***7*** | 0.775 | | 0.815 | 0.817 | 0.781 | 0.794 | 0.796 | 0.808 | 0.802 | 0.798 | 0.798 |
|  | ***8*** | 0.792 | | 0.793 | 0.817 | 0.796 | 0.781 | 0.791 | 0.808 | 0.750 | 0.791 | 0.791 |
|  | ***9*** | 0.788 | | 0.792 | 0.798 | 0.802 | 0.798 | 0.781 | 0.773 | 0.765 | 0.787 | 0.787 |
| **column mean** | | 0.767 | | 0.799 | 0.798 | **0.804** | 0.792 | 0.800 | 0.796 | 0.786 | 0.767 |  |

**
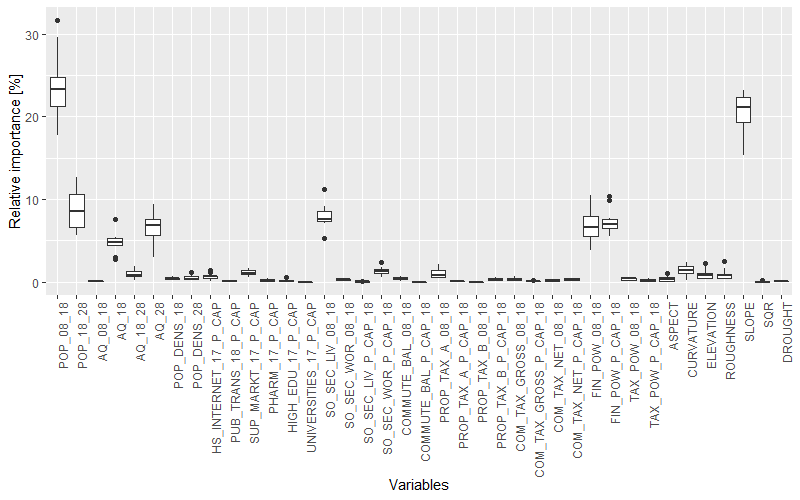
**

**Fig. S7: Relative importance of explanatory variables distinguishing booming (cluster 2) from transition regions (cluster 3) (10 folds each); explained variance: 91.00 %**

**
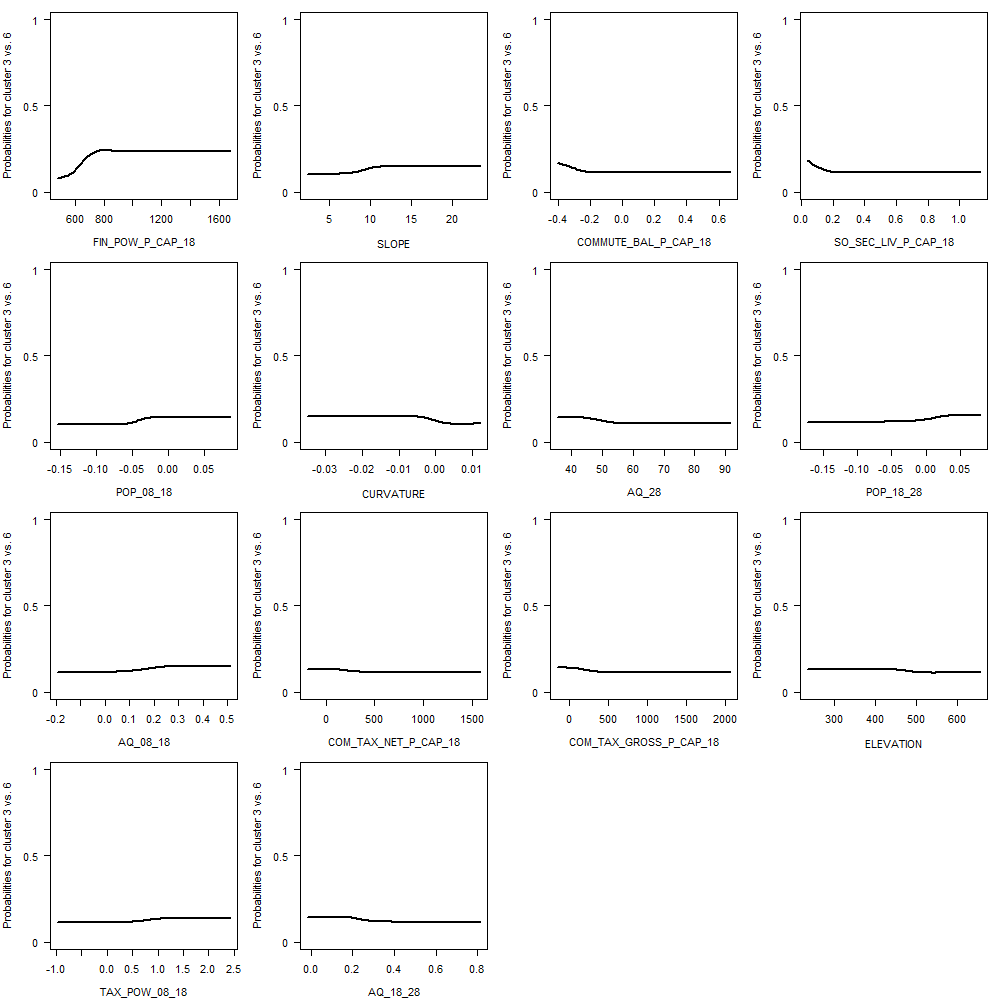
**

**Fig. S8: Partial dependency plots for more influential explanatory variables (descending order of importance) distinguishing transition (cluster 3) (probability towards 1) from moderate regions (cluster 6) (probability towards 0).**

**
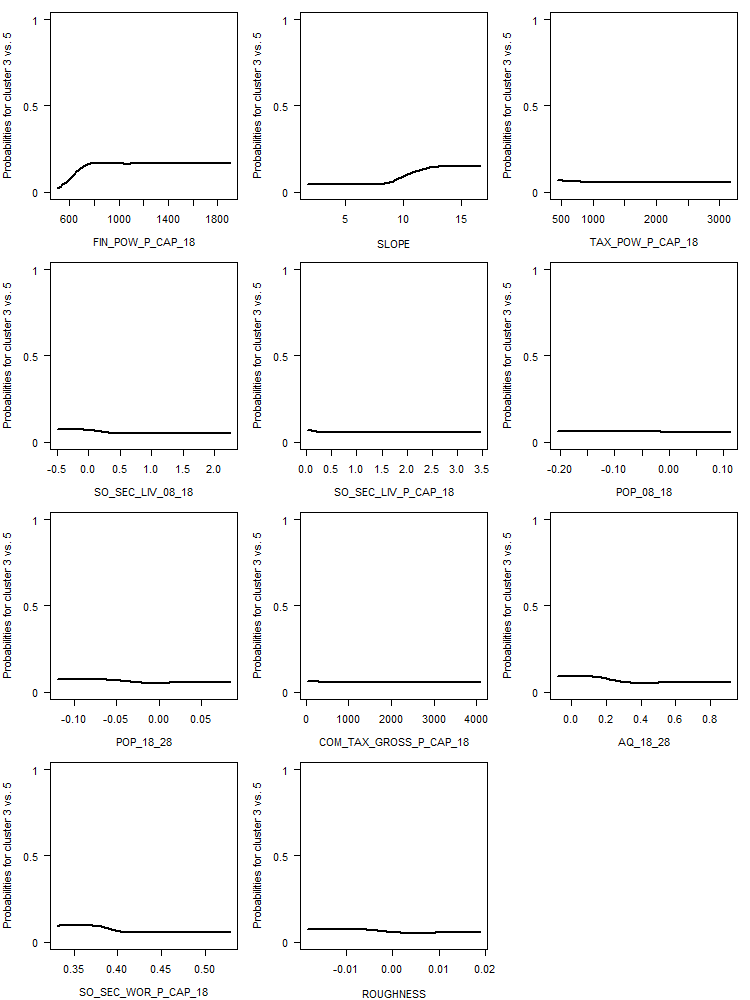
Fig. S9: Partial dependency plots for more influential explanatory variables (descending order of importance) distinguishing transition (cluster 3) (probability towards 1) from prosperous regions (cluster 5) (probability towards 0).**

**
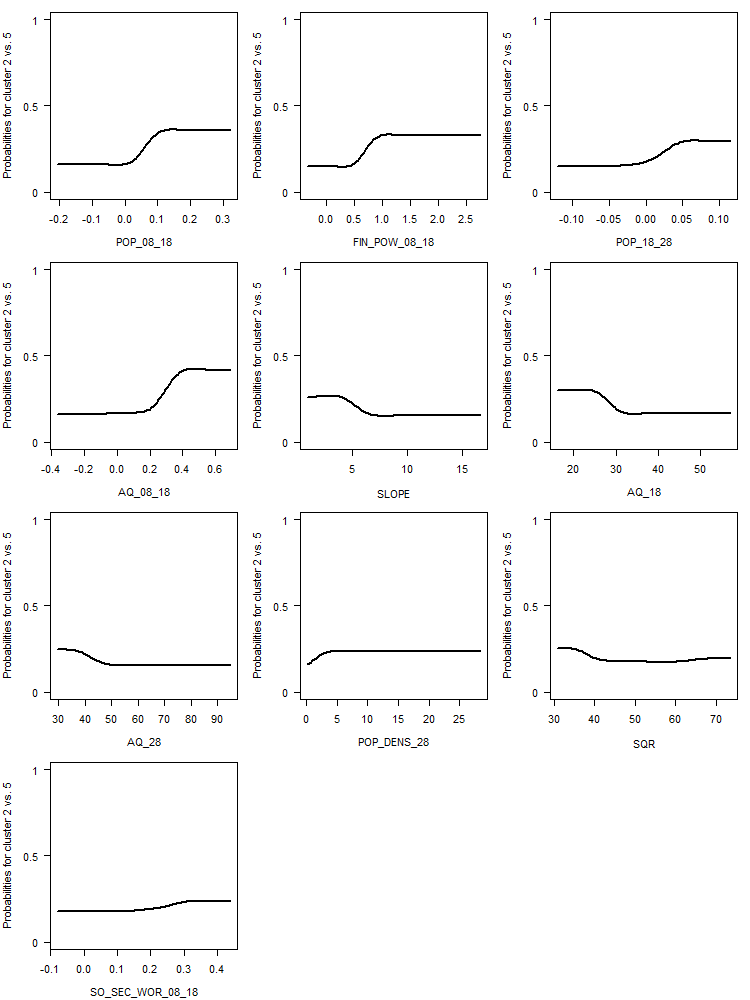
Fig. S10: Partial dependency plots for more influential explanatory variables (descending order of importance) distinguishing booming (cluster 2) (probability towards 1) from prosperous regions (cluster 5) (probability towards 0).**

**
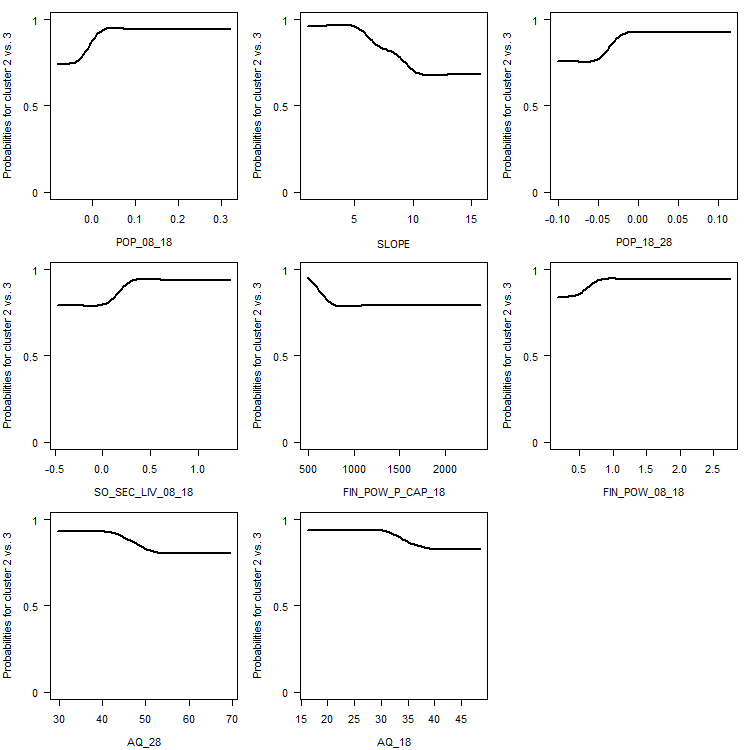
Fig. S11: Partial dependency plots for more influential explanatory variables (descending order of importance) distinguishing booming (cluster 2) (probability towards 1) from transition regions (cluster 3) (probability towards 0).**

**
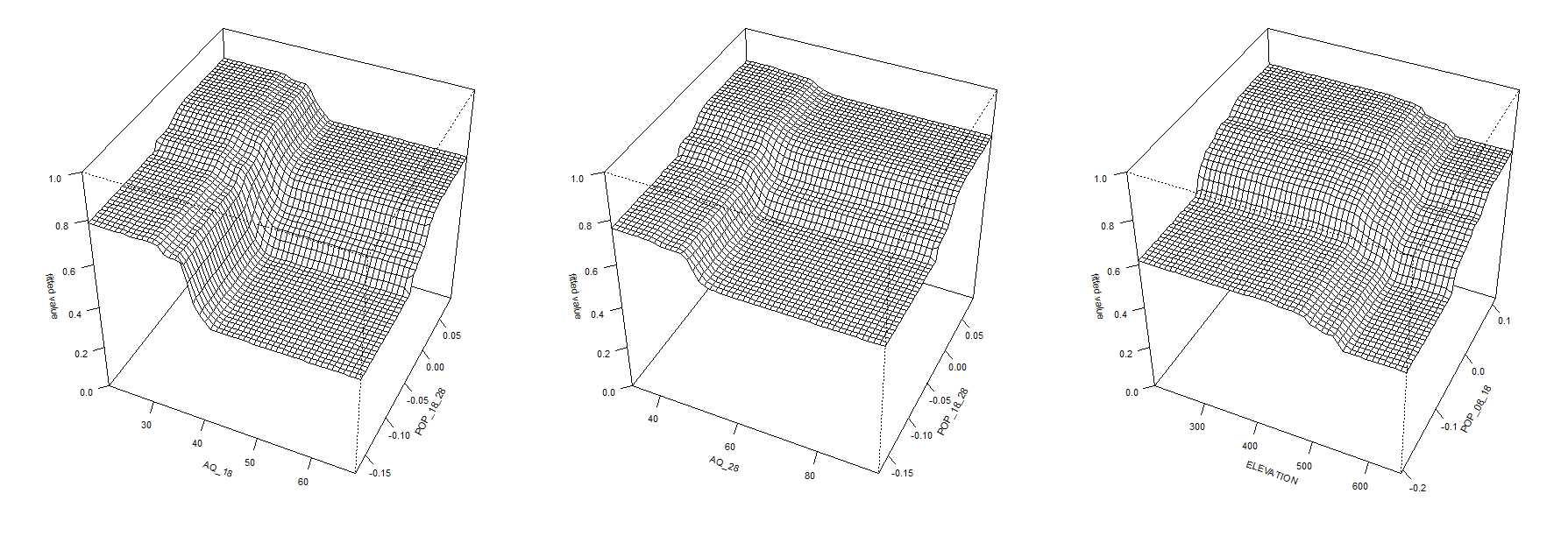
**

**Fig. S12: Major interactions of explanatory variables distinguishing prosperous (cluster 5) (probability towards 1) from moderate regions (cluster 6) (probability towards 0).**

**
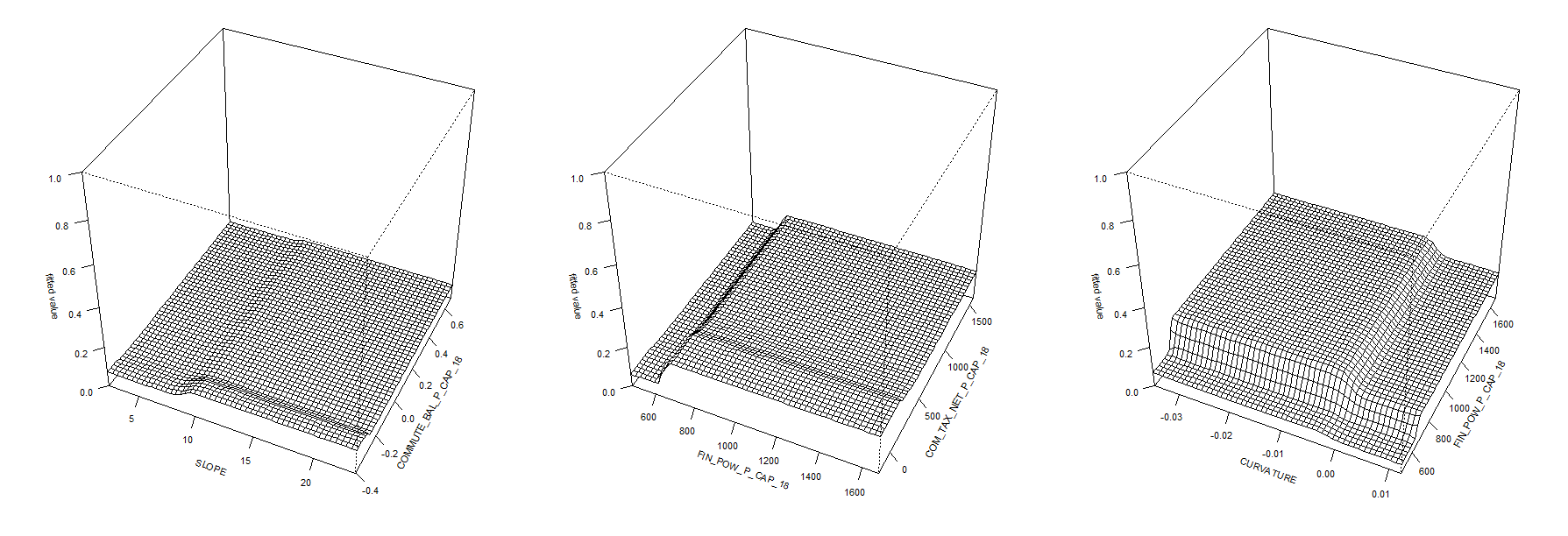
**

**Fig. S13: Major interactions of explanatory variables distinguishing transition (cluster 3) (probability towards 1) from moderate regions (cluster 6) (probability towards 0).**

**
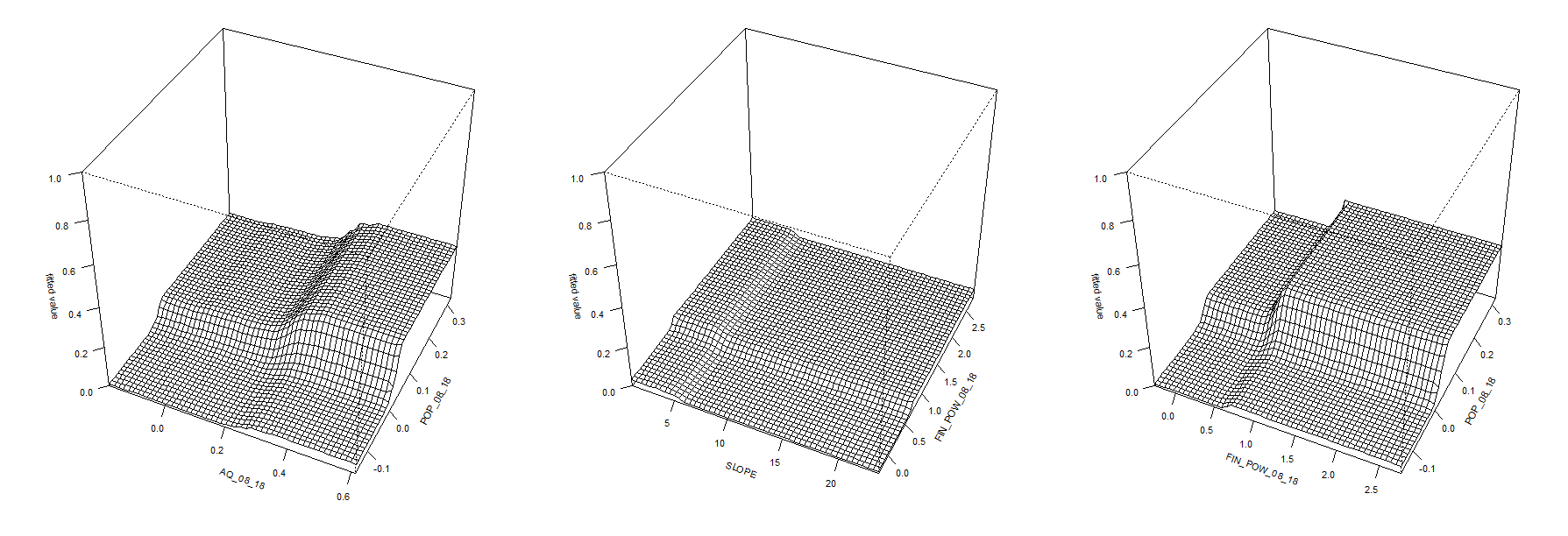
**

**Fig. S14: Major interactions of explanatory variables distinguishing booming (cluster 2) (probability towards 1) from moderate regions (cluster 6) (probability towards 0).**

**
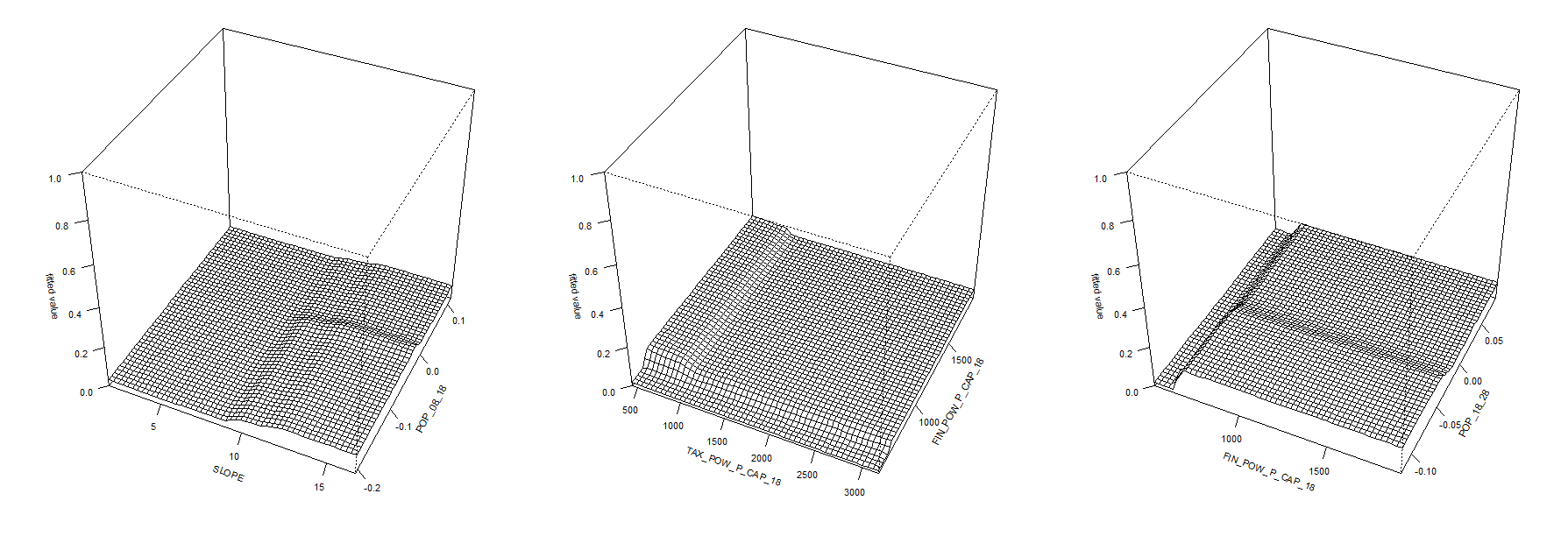
**

**Fig. S15: Major interactions of explanatory variables distinguishing transition (cluster 3) (probability towards 1) from prosperous regions (cluster 5) (probability towards 0).**
